# Integrated analysis, transcriptome-lipidome, reveals the effects of *INO-*level (*INO2 *and *INO4*) on lipid metabolism in yeast

**DOI:** 10.1186/1752-0509-7-S3-S7

**Published:** 2013-10-16

**Authors:** Pramote Chumnanpuen, Intawat Nookaew, Jens Nielsen

**Affiliations:** 1 Department of Zoology, Faculty of Science, Kasetsart University, Bangkok, Thailand; 2 Systems and Synthetic Biology, Department of Chemical and Biological Engineering, Chalmers University of Technology, Gothenburg, Sweden

## Abstract

**Background:**

In the yeast *Saccharomyces cerevisiae*, genes containing UAS_INO _sequences are regulated by the Ino2/Ino4 and Opi1 transcription factors, and this regulation controls lipid biosynthesis. The expression level of *INO2 *and *INO4 *genes (*INO*-level) at different nutrient limited conditions might lead to various responses in yeast lipid metabolism.

**Methods:**

In this study, we undertook a global study on how *INO*-levels (transcription level of *INO2 *and *INO4*) affect lipid metabolism in yeast and we also studied the effects of single and double deletions of the two *INO*-genes (deficient effect). Using 2 types of nutrient limitations (carbon and nitrogen) in chemostat cultures operated at a fixed specific growth rate of 0.1 h-1 and strains having different *INO*-level, we were able to see the effect on expression level of the genes involved in lipid biosynthesis and the fluxes towards the different lipid components. Through combined measurements of the transcriptome, metabolome, and lipidome it was possible to obtain a large dataset that could be used to identify how the *INO*-level controls lipid metabolism and also establish correlations between the different components.

**Results:**

In this study, we undertook a global study on how *INO*-levels (transcription level of *INO2 *and *INO4*) affect lipid metabolism in yeast and we also studied the effects of single and double deletions of the two *INO*-genes (deficient effect). Using 2 types of nutrient limitations (carbon and nitrogen) in chemostat cultures operated at a fixed specific growth rate of 0.1 h-1 and strains having different *INO*-level, we were able to see the effect on expression level of the genes involved in lipid biosynthesis and the fluxes towards the different lipid components. Through combined measurements of the transcriptome, metabolome, and lipidome it was possible to obtain a large dataset that could be used to identify how the *INO*-level controls lipid metabolism and also establish correlations between the different components.

**Conclusions:**

Our analysis showed the strength of using a combination of transcriptome and lipidome analysis to illustrate the effect of *INO*-levels on phospholipid metabolism and based on our analysis we established a global regulatory map.

## Background

Phospholipid synthesis in the yeast *Saccharomyces cerevisiae *is a complex process that involves regulation by both genetic and biochemical mechanisms [1-3]. The activity levels of phospholipid synthesis enzymes are controlled by gene expression, e.g., transcription, and by other factors, i.e., lipids, water-soluble phospholipid precursors and products, and covalent modification by phosphorylation.

At the transcription level, the heterodimeric Ino2/Ino4 activator and the transcription factor Opi1 are global regulators affecting the expression of a large number of phospholipid biosynthetic genes [4-6]. Opi1, containing a leucine zipper motif, has been known as a negative regulator of phospholipid biosynthesis and it can also repress the transcription of *INO2 *and *INO4 *[7,8]. In the absence of Opi1, the transcriptional level of both *INO2 *and *INO4 *(so called "***INO*-level**") will be up-regulated compared to the reference strain. Ino2p and Ino4p binds to so-called inositol-choline response elements (ICRE), and ICRE-bound Ino2p can interact with coactivator complexes such as Snf1 kinase that has histone kinase function, the SAGA complex, and the TFIIB complex when *OPI1 *is disrupted or not presented in the nucleus [9,10]. Opi1 is also necessary for repression of ICRE-dependent transcription when inositol-choline is present in excess [8,11]. Under this condition, Opi1 is localized in the nucleus [12] and prevents Ino2 from activation of target genes by recruiting the pleiotropic co-repressors such as the Cyc8/Tup1 complex [13,14] or Sin3p [15-19]. Consequently, Ino2 variants (which are defective in interacting with Opi1) lead to the repression of phospholipid synthetic genes [20]. However, a genome wide transcription analysis of the effect of varying *INO*-levels that effect lipid metabolism has never been studied before.

It is known that the transcription of the phospholipid biosynthetic genes is maximally repressed in the presence of the phospholipid precursors inositol and choline (IC) [4,21,22]. A highly conserved 10bp-element (5'-CATGTGAAAT-3'), in at least one copy is found in the promoters of the lipid co-regulated genes such as *INO1*, *CHO1*, *CHO2*, *OPI3*, *FAS1*, *FAS2*, *ACS2*, and *ACC1 *[23-28]. This element has been shown to be both necessary and sufficient for the IC response, the so called "inositol/choline-responsive element" or "inositol-sensitive upstream activating sequence" (ICRE or UAS_INO _motifs)[23,24,29]. These motifs are bound by a heterodimer of positive regulators Ino2p and Ino4p containing a basic helix-loop-helix (bHLH) structural motif [21,30-32] which is necessary and sufficient for dimer formation and specific interaction with the UAS_INO _motif [10,32,33]. Recent studies have shown that expression of several genes, probably unrelated to phospholipid metabolism, is also affected by Ino2p and Ino4p [22,29,34]. Importantly, over-expression of *INO2 *(but not of *INO4*) counteracts IC repression, suggesting Ino2p as a possible target of the signal transduction pathway triggering IC repression [35,36]

At the biochemical mechanism level, the level of phosphatidic acid (PA) is controlled by the biochemical regulation of key phospholipid synthesis enzymes [12] and it plays a central role in the regulation of phospholipid synthesis gene expression [1,33,37].

In this study, we undertook a global study of lipid metabolism in response to different *INO*-levels (*INO2 *and *INO4*): (1) normal *INO*-level using wild-type (CEN.PK113-7D), (2) high *INO*-level using an *opi1*Δ strain, and (3) low (or rather absent) *INO*-level using an *ino2*Δ *ino4*Δ double deletion strain. Moreover, we also focused on the deficient factors as a sub story comparing the individual knockout strains (*ino2*Δ and *ino4*Δ) with the double deletion strain. Using a systems biology approach [38] a global regulatory model for lipid metabolism could be established. With 2 types of nutrient limitations (carbon and nitrogen) and different *INO*-level, we were able to see the effect on expression level of the genes involved in lipid biosynthesis and the fluxes towards the different lipid components. Through combined measurements of the transcriptome, metabolome, and lipidome it was possible to obtain a large dataset that could be useful to identify the effect of *INO*-level and also establish correlations between the different components.

## Methods

### Materials

All chemicals were reagent grade. Phospholipids, fatty acid methyl ester and neutral lipids standards were purchased from Sigma.

### Agar spot test on SD media with different inositol concentrations

The reference strain CEN.PK 113-7D and 5 mutants as shown in table 1 were grown on SD agar plate (containing Yeast Nitrogen Base without amino acids and inositol, Formedium LTD, England) for 48 hours. The SD agar plates were supplemented with 0, 1.39, 75 or 220 µM of inositol.

**Table 1:** List of strains used in this study and their genotypes

Strain	Genotype	Remark
CEN.PK113-7D	*MAT*a *URA3 HIS3 LEU2 TRP1 SUC2*	*Reference*
CEN.PK1029-1A	*MAT*a *URA3 HIS3 LEU2 TRP1 SUC2 opi1Δ::loxP-Kan-loxP*	*opi1Δ *(high *INO*)
CEN.PK1033-9A	*MAT*a *URA3 HIS3 LEU2 TRP1 SUC2 ino2Δ::loxP-Kan-loxP ino4Δ::loxP-Kan-loxP*	*ino2Δino4Δ *(low *INO*)
CEN.PK1027-1B	*MAT*a *URA3 HIS3 LEU2 TRP1 SUC2 ino2Δ::loxP-Kan-loxP*	*ino2Δ*
CEN.PK1028-2A	*MAT*a *URA 3 HIS3 LEU2 TRP1 SUC2 ino4Δ::loxP-Kan-loxP *	*ino4Δ *

### Strains, cultivation, and fermentation profile

The S. cerevisiae strains used in this study were a prototrophic strain CEN.PK 113-7D (Mat**a **Mal2-8c SUC2) [39] and its derivative (opi1∆, ino2∆, ino4∆, and ino2∆ino4∆) supplied by Peter Kötter (Frankfurt, Germany). All strains in this study were prototrophic and with mating type a (Table 1). Steady-state aerobic chemostat cultures were grown at 30 ˚C in 1.2 L bioreactors (DASGIP, Germany) with working volume of 0.5 L using a dilution rate of 0.10 (±0.005) h-1. For the C-limited and N-limited cultures, the medium composition was the same as in a previous study [40,41] which contained 75 μM of inositol and 1mM of choline [24,42]. The pH was controlled at 5.00 ± 0.05 with 2M KOH and dissolved oxygen was kept above 30%. Chemostat cultivation ensured that metabolic and regulatory changes observed were specific to the INO-level and also the disruptions of INO2 and/or INO4, and not complicated by external effects resulting from different specific growth rates.

Samples were harvested from the cultivation media every second hour and immediately filtered through a 0.45 μm pore-size cellulose acetate filter (VWR) and stored at -20 ˚C until analysis. Biomass production was evaluated by measuring of optical density (OD_600_) and dry cell weight. Glucose, glycerol, ethanol, and acetate concentrations were determined by HPLC analysis using an Aminex HPX-87H column (Biorad, Hercules, CA) [43].

### Transcriptome analysis

#### Transcriptome data acquisition

Samples for RNA extraction were taken after 50 h (i.e. 5 retention times) of steady-state by rapidly taking 20 ml of culture and mixing with 30 ml of crushed ice in a 50 ml Falcon tube to cool down the samples immediately. The cells were harvested by centrifuging at 4000 rpm and 2˚C for 3 min, and then frozen in liquid nitrogen and stored at -80˚C until subsequent RNA extraction. The cells were mechanically disrupted using FastPrep homogenizer (MP Biomedicals) and total RNA was isolated using the RNeasy Mini Kit (QIAGEN). The quality of total RNA was assessed using an Agilent 2100 Bioanalyzer (Agilent Technologies) with RNA 6000 Nano LabChip kit (Agilent Technologies). The labeled RNA was synthesized using the GeneChip 3' IVT Express Kit (Affymetrix), which was then hybridized onto the GeneChip Yeast Genome 2.0 Arrays (Affymetrix). Staining and washing of the hybridized arrays were carried out on the GeneChip^® ^Fluidics Station 450 (Affymetrix) and scanned using the GeneChip Scanner 300 7G (Affymetrix). All transcriptome data of this study can be found at Gene Expression Omnibus with accession number GSE36298.

#### Transcriptome data analysis

The transcriptome data were analyzed using Bioconductor in R. Raw data were normalized and processed together with Probe Logarithmic Intensity Error (PLIER http://media.affymetrix.com/support/technical/technotes/plier_technote.pdf). Pairwise T-test analysis was performed to determine the genes whose expression level is significantly changed due to INO-level, as well as the sufficiency factor. The calculated P-values were corrected for multiple testing by FDR method. A cut-off value of adjusted P value<0.01 was set to assess statistical significance.

### Lipid data acquisition

#### Total lipid extraction

The lipid extraction method was adapted from Bligh and Dyer[44]. First, 15 mg of freeze-dried cell pellets were treated with 1 unit μl-1 of zymolyase digesting buffer (1.2 M glycerol, 100 mM sodium thioglycolate, 50 mM Tris-sulfate, pH 7.5) at 37°C for 15 min, followed by centrifugation at 3000 rpm for 3 min to collect the spheroplast, which was mixed with internal standards (heptadecanoic acid and glyceryl tri-heptadecanoate, 25 µg of each). After the addition of 7 ml of chloroform-methanol (2:1, v/v), the mixture was shaken horizontally at 300 rpm 4°C for 3 h, mixed with 1.7 ml of sodium chloride solution (0.73%) and centrifuged at 3000 rpm 4°C for 4 min for phase separation. The lower (organic)-phase was collected and the remaining was re-extracted with 5 ml of chloroform-methanol (85:15 v/v). The lower (organic)-phase was collected and pooled with the previous organic fraction and kept at -20°C until further analysis.

#### Lipid class separation, identification, and quantification using HPLC-CAD

Lipid separation and quantification were performed using our developed method [45]. Lipid separation was accomplished by HPLC (Dionex) equipped with charge aerosol detector; CAD (Corona) and the gas connected was nitrogen gas with 35 psi gas pressure. All the separated fractions were then collected by automated fraction collector; AFC-3000 (Dionex). A 20 μl volume of sample was injected in to the Luna 5 µm HILIC 200 Å 100 × 3.0 mm LC Column (Phenomenex). The flow-rate was 0.8 ml/min and the column temperature was kept at 25˚C during all runs. The chromatogram was record at 10 Hz frequency and gain for 100 pA. The polar and neutral lipid classes were separated by three solvent mixtures and gradient systems as follow: (A) hexane-acetic acid (99:1, v/v); (B) acetone-isopropanol-acetic acid (29:70:1, v/v/v); (C) water-acetone-isopropanol-acetic acid (9:20:70:1, v/v/v/v). Triethylamine (0.08%, v/v) was added to the solvent C to adjust pH. The samples were injected at time 0 and the gradient profile started at 100% of solvent A and the solvent B was gradually increased to 5% in 14 min and it was always kept at 5% along the process. At 15 min time point, solvent C was slowly entering to the system and rising up to 40% in 5 min. Then solvent C was slowly increased until 45% in 20 min. Finally, the gradient was reduced from 5% to 0% of solvent B and from 45% to 0% of solvent C in 5 min and then maintained at 100% of solvent A for 5 min. In total, the solvent program for the separation of all lipid classes took 45 min.

#### Identification and quantification

Pure lipid standards were analyzed individually using chromatography to confirm their retention times and purity. Lipid standards were also co-eluted together with samples to identify peaks in unknown samples. Solutions of known concentrations of different lipid classes were mixed and lipid standard curves were generated to study the linearity of the detection method and to quantify lipid classes in unknown samples. Calibration curves were prepared for 5-1000 μg ml-1 of PA, PE, PC, PS, PI, ES, TAG, FA, and ES. Each concentration of the standard solutions was injected twice and the average log_10 _peak area for each lipid was plotted against the absolute amount of lipid. Correlation (r2) was determined for all curves by linear regression.

#### Fatty acid methylesters (FAMEs) analysis

To quantify the distribution of fatty acid long chain species, we used standard procedure developed in our laboratory which is based on the previous protocol by Khoomrung et al. [46]. Briefly, 10 mg of freeze-dried samples was mixed with 4 mL of hexane, 2 mL of 14% BF_3 _(in Methanol) and 5 µg of internal standard (17:0) fatty acid standard was added. The sample was then flushed into the tube's head space with nitrogen gas for 30s and closed tightly with a Teflon screw cap. The tube was placed in a vessel containing 30 mL of milliQ water and then sealed with TFM screw cap. The tube was heated using microwave digestion system (milestone start D, Sorisole Bergamo, Italy) equipped with rotor PRO-24. The temperature programming of microwave digestion was ramped (from room temperature) to 120 ˚C within 6 min and maintained for 10 min. After cooling down sample at the room temperature, 2 mL of milliQ water was added and shaken vigorously for 1 min and centrifuged at 2500 rpm for 5 min. The upper phase (hexane phase which contained the FAMEs) was analysed by GC-MS.

The FAMEs were separated and quantified using Focus GC ISQ single quardrupole GC-MS (Thermo Fisher scientific, Germany). The separation of FAMEs was performed on Zebron (ZB-WAX) GC column (30 m × 0.25 mm I. D., 0.25 μm film thickness) from Phenomenex, Macclesfield, UK. Sample was injected in splitless injection mode (1µL at 240 ˚C) and Helium was a carrier gas (1 mL/min). The column temperature was initially set at 50 °C (1.5 min), then temperature was ramped to 180 °C (25°C/min) for 1 min, then increased to 220 °C (10°C/min) and held for 1 min. Finally, temperature was increased to 250 °C (15°C/min) and held for 3.0 min. Mass transfer line and ion source were set at 250 °C and 200 °C, respectively. The FAMEs were detected with electron ionization (70 eV) in scan mode (50-650 m/z) and selected ion monitoring mode at m/z 55, 67, 74 and 79 (for quantitative analysis). The identification of unknown FAMEs was achieved by comparing their retention times and mass spectrum profiles with known standards (Sigma-Aldrich, USA). The quantification of FAMEs was performed using QuanBrowser function in Xcalibur software version 2.0 (Thermo Fisher Scientific). According to the serial dilution of FAME mix standards and were normalized according to the internal standard fatty acid C17:0. The average molecular weights of each PL (Table.S1 in additional file 1) were used for mg/gDW and mmol/gDW units conversion (were later used for metabolic fluxes analysis).

### Integrated analysis

The statistical adjusted P-values of each hypothesis testing were overlaid on the three networks graph of Gene Ontology, Transcription factor-gene interaction and genome-scale metabolic model iIN800 [47] (metabolite-gene interaction). Then reporter algorithm [48] was performed to obtain significant values (reporter p-value) of GO terms, Transcription factors (TF) and metabolites. The Platform for integrative analysis of omics data (PIANO) package for R ([49]available at http://www.bioconductor.org/packages/2.13/bioc/html/piano.html) has been performed for integrated analysis. All the features presented in heatmaps are those features that have reporter P-value < 0.001 and P-value < 0.01 were considered for INO-level and deficient factors, respectively.

## Results

### Inositol is essential in the low *INO*-level yeast

**Figure 1:**
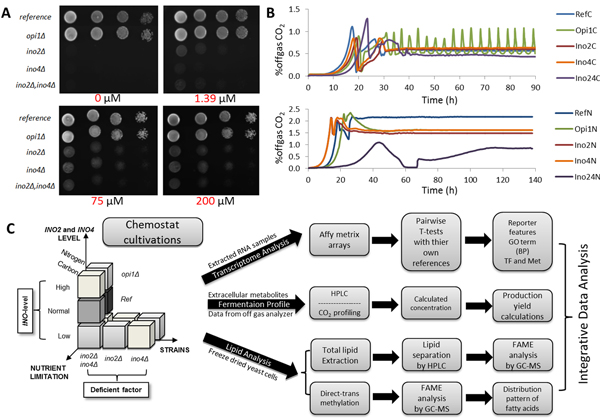
**Procedures of integrated analysis to reveal the effects of *INO-*level (*INO2 *and *INO4*) on lipid metabolism in yeast**. Spot test of all strains on SD agar plates with different inositol concentrations (A). Off gas CO_2 _profile of each mutant strain during the chemostat cultivation at C-limitation and N-limitation (B). Schematic analysis of a workflow for integrated analysis, transcriptome-lipidome, of *INO*-level (key factor) and deficient factor effect on lipid biosynthesis (C).

To estimate the required inositol concentration for low *INO*-level (either *ino2Δ*, or *ino4Δ*, or double deletion of them), a spot test with the 5 yeast strains on different concentrations of inositol were performed (Figure 1A). Since the Ino2-Ino4 heterodimer regulates positively the expression of *INO1 *(a structural gene for inositol-1-phosphate synthase which is required for inositol synthesis) and other phospholipid genes containing UAS_INO _element [31,50], deletion of *INO2 *and *INO4 *results in a requirement for supplementation of myo-inositol. However, myo-inositol is included in our minimal medium in a concentration of 250 µg/L (corresponding to about 1.39 µM) [41]. The spot test showed that *opi1Δ *as well as reference strain are prototroph for inositol but at least 75 µM of inositol is sufficient for low *INO*-level strains (either single or double deletion of *INO2 *and *INO4*) to grow. However, higher concentrations of inositol (even 200 µM) did not make much difference in term of growth. Based on this we conclude that the low *INO*-level strains are auxotroph for inositol, and this leads us to an experimental design using 75 µM of inositol in the medium for all the chemostat cultures.

### Cell physiology and carbon metabolism

To see the effects on cell growth of each mutant strain, 5 strains (the reference, *opi1Δ*, *ino2Δino4*, *ino2Δ*, *ino4Δ*) were grown in batch cultivations using a defined minimum medium and switched to chemostat immediately after glucose was depleted. Table 2 summarizes the basic physiological parameters for growth on glucose for all the strains in either C-limited or N-limited conditions. During the batch, *ino2*Δ *ino4*Δ double deletion had the highest maximum specific growth rate in the C-limited medium but the lowest maximum specific growth rate in the N-limited medium (Figure 1B). However, it was clear that the double deletion strain had longer lag phase during batch cultivation in both C-limited and N-limited conditions (25h and 60h, respectively). Interestingly, during the chemostat cultivation we found this double deletion strain had the lowest biomass yields compared to the other strains. Interestingly, we found that the *opi1Δ *strain exhibited metabolic oscillations in the glucose limited chemostat cultures. We therefore took the samples for transcriptome analysis at two time points, at the maximum and minimum level of CO_2 _production.

**Table 2:** Physiological parameters of reference and mutants in chemostat cultivation

Strains	µ_max_^1^	Left Glc (g/L)	RQ	Y_SX_^2^	Y_SE_^3^	Y_SG_^4^	Y_SA_^5^	Y_SS_^6^	Y_SC_^7^
**RefC**	0.384 ± 0.001	0.015 ± 0.000	0.817 ± 0.007	0.539 ± 0.010	n.d.	0.019 ± 0.001	n.d.	n.d.	0.124 ± 0.003
**Opi1CT**	0.343 ± 0.001	0.009 ± 0.001	1.058 ± 0.020	0.557 ± 0.002	0.004 ± 0.000	0.019 ± 0.000	0.003 ± 0.000	n.d.	0.208 ± 0.004
**Opi1CB**	0.343 ± 0.001	0.009 ± 0.001	0.819 ± 0.003	0.550 ± 0.001	n.d.	0.019 ± 0.000	n.d.	n.d.	0.106 ± 0.003
**Ino2C**	0.340 ± 0.000	0.018 ± 0.001	0.805 ± 0.011	0.597 ± 0.020	n.d.	0.018 ± 0.001	0.003 ± 0.000	n.d.	0.129 ± 0.006
**Ino4C**	0.369 ± 0.001	0.010 ± 0.000	0.837 ± 0.009	0.571 ± 0.007	n.d.	0.018 ± 0.002	n.d.	n.d.	0.135 ± 0.002
**Ino24C**	0.436 ± 0.000	0.039 ± 0.001	0.889 ± 0.013	0.362 ± 0.028	0.098 ± 0.011	0.022 ± 0.003	0.019 ± 0.000	0.002 ± 0.000	0.101 ± 0.002
**RefN**	0.486 ± 0.000	20.387 ± 1.667	1.286 ± 0.008	0.128 ± 0.004	0.485 ± 0.048	0.008 ± 0.000	0.007 ± 0.001	0.002 ± 0.000	0.124 ± 0.004
**Opi1N**	0.499 ± 0.001	15.521 ± 1.809	1.260 ± 0.001	0.096 ± 0.003	0.316 ± 0.017	0.005 ± 0.000	0.005 ± 0.000	n.d.	0.075 ± 0.017
**Ino2N**	0.495 ± 0.001	28.286 ± 0.877	1.159 ± 0.008	0.168 ± 0.002	0.305 ± 0.015	0.009 ± 0.000	0.006 ± 0.001	n.d.	0.102 ± 0.005
**Ino4N**	0.494 ± 0.001	28.837 ± 0.427	1.261 ± 0.017	0.164 ± 0.003	0.337 ± 0.010	0.008 ± 0.000	0.006 ± 0.000	n.d.	0.103 ± 0.001
**Ino24N**	0.410 ± 0.000	35.944 ± 1.867	1.269 ± 0.010	0.058 ± 0.008	0.370 ± 0.026	0.017 ± 0.001	0.038 ± 0.001	n.d.	0.076 ± 0.005

Note: All values are average ± SD from three biological replicates.

^1 ^maximum specific growth rate on glucose during batch cultivation (with 10 g/L and 60 g/L initial glucose for C-limited and N-limited conditions, respectively)

^2 ^Biomass yield on glucose in chemostat cultures (g biomass formed/g glucose consumed)

^3 ^Ethanol yield on glucose in chemostat cultures (g ethanol formed/g glucose consumed)

^4 ^Glycerol yield on glucose in chemostat cultures (g glycerol formed/g glucose consumed)

^5 ^Acetate yield on glucose in chemostat cultures (g acetate formed/g glucose consumed)

^6 ^Succinate yield on glucose in chemostat cultures (g succinate formed/g glucose consumed)

^7 ^CO_2 _yield on glucose in chemostat cultures (mg CO2 formed/g glucose consumed)

These two samples represent reductive-building (R/B) and reductive charging (R/C) of the yeast metabolic cycle respectively [51] and the transcriptional data analysis for both of them were performed to see the differences from 2 metabolic phases (Fig. S1 in additional file 1). The heatmap of reporter shows that the differences between these 2 metabolic phases mainly affects genes associated with differences in cell cycles period. According to the respirative quotient (RQ) from Table 2, the phase of the population at maximum CO_2 _production showed about 20% higher RQ compared with all the other strains at C-limitation. Based on this we therefore decided to exclude this sample and only use the sample from the minimum CO_2 _production as a high *INO*-level representative at C-limitation condition.

Double deletion of Ino2 and Ino4 resulted in a substantial reduction (about 35% in C-limited and 55% in N-limited conditions) in biomass yield compared with the reference strain (Table 2). Interestingly, deletion of Opi1 resulted in a small reduction (approx. 25%) in biomass only at N-limited but not at C-limited conditions.

### *INO*-level in nutrient-limited conditions

**Figure 2:**
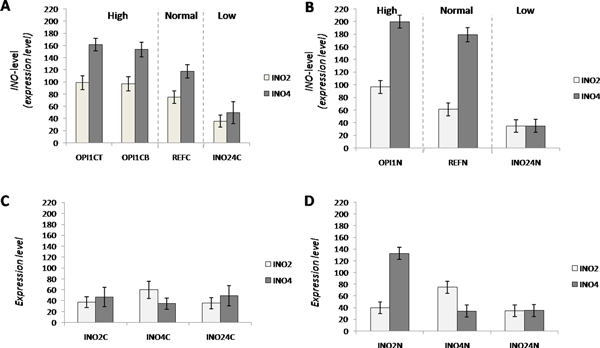
**The normalized expression values of *INO2 and INO4 *(*INO*-level) for each strain**. The key factor set shows the different *INO*-level at C-limitation (A) and N-limitation (B). The deficient factor group set shows the different *INO*-level at C-limited (C) and N-limited (D)

The levels of *INO*-gene (*INO2 *and *INO4*) expression in both C-limited and N-limited conditions were investigated according to the expression level from micro array results (Figure 2). Even though there were metabolic cycle patterns with the *opi1*Δ strain grown at C-limited condition, the expressions of the *INO *genes were consistently high (Figure 2A). This evidence shows that *INO *genes are up-regulated when their repressor, Opi1, is absent. At C-limited condition, INO levels are low in both the two single and the double deletion strains (Figure 2A and 2C). Interestingly, the *INO4 *gene seems to be expressed at a high level at N-limitation (Figure 2B) and it possibly also tries to compensate the failure of *INO2 *expression by up-regulating its expression in the *ino2*Δ strain at N-limitation (Figure 2D).

### Global transcriptome changes due to *INO*-level in nutrient-limited conditions

**Figure 3:**
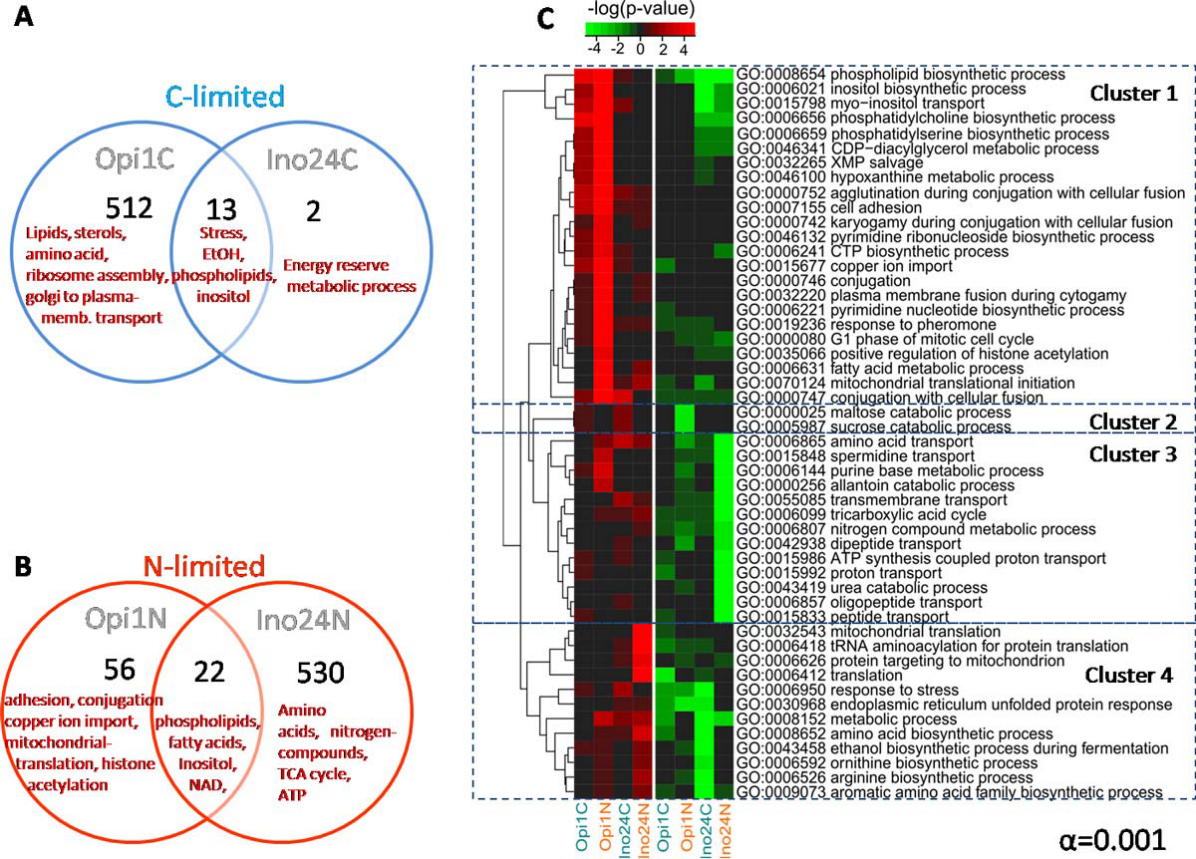
**Transcriptional data analysis of 3 different *INO*-level strain**. Venn's diagram of significant genes of mutant strains caused by different *INO*-level when focus on C-limitation (A) and N-limitation (B) separately, *p*-value < 0.001 were considered. (C) A heat map of overrepresented GO terms (Biological Process) of each factor comparison showing in the range of -4 to 4 of log(*p*-value). The green color indicates down-regulation and red indicates up-regulation compared to the reference strain.

We used the Affymetrix DNA microarray platform to measure the expression level of all genes and access the global effect caused by the *INO*-level under nutrient-limited conditions (C-limited and N-lim). The transcriptome data were decomposed using principal component analysis (PCA) and Student's T-test analysis (α = 0.001). The transcriptome data are presented in Venn diagrams at C-limited (Figure 3A) and at N-limited (Figure 3B) showing that the high *INO*-level (*opi1*Δ) strain had more genes being significantly changed at C-limitation. On the other hand, the low *INO*-level (*ino2Δino4Δ *double deletion) strain had more genes being significantly changed at N-limitation.

From clustering of reporter GO terms (biological process at *P *< 0.001), 4 main clusters were identified (Figure 3C). Cluster 1 which is the largest group contains the genes involving the phospholipid biosynthesis, myo-inositol biosynthesis and transport, fatty acid metabolic process, cell conjugation, and ribonucleoside biosynthetic process which were highly up-regulated due to the deletion of *OPI1 *(high *INO*-level), especially at N-limited condition, but down-regulated at low *INO*-level at both C- and N-limitation. Cluster 2 contains genes involving maltose and sucrose catabolic process which were highly up-regulated due to the C-limitations. Cluster 3 contains genes involving nitrogen compound metabolism, amino acid and peptide transport, and proton transport which were down-regulated in the double mutant at N-limited condition. Cluster 4 contains genes involving amino acid biosynthesis, mitochondria biogenesis, and endoplasmic reticulum associated unfolded protein responses (ER-UPR) which were highly significant in the double mutant strain. These genes were up-regulated at low *INO*-level and N-limitations but down-regulated at C-limitation.

**Figure 4:**
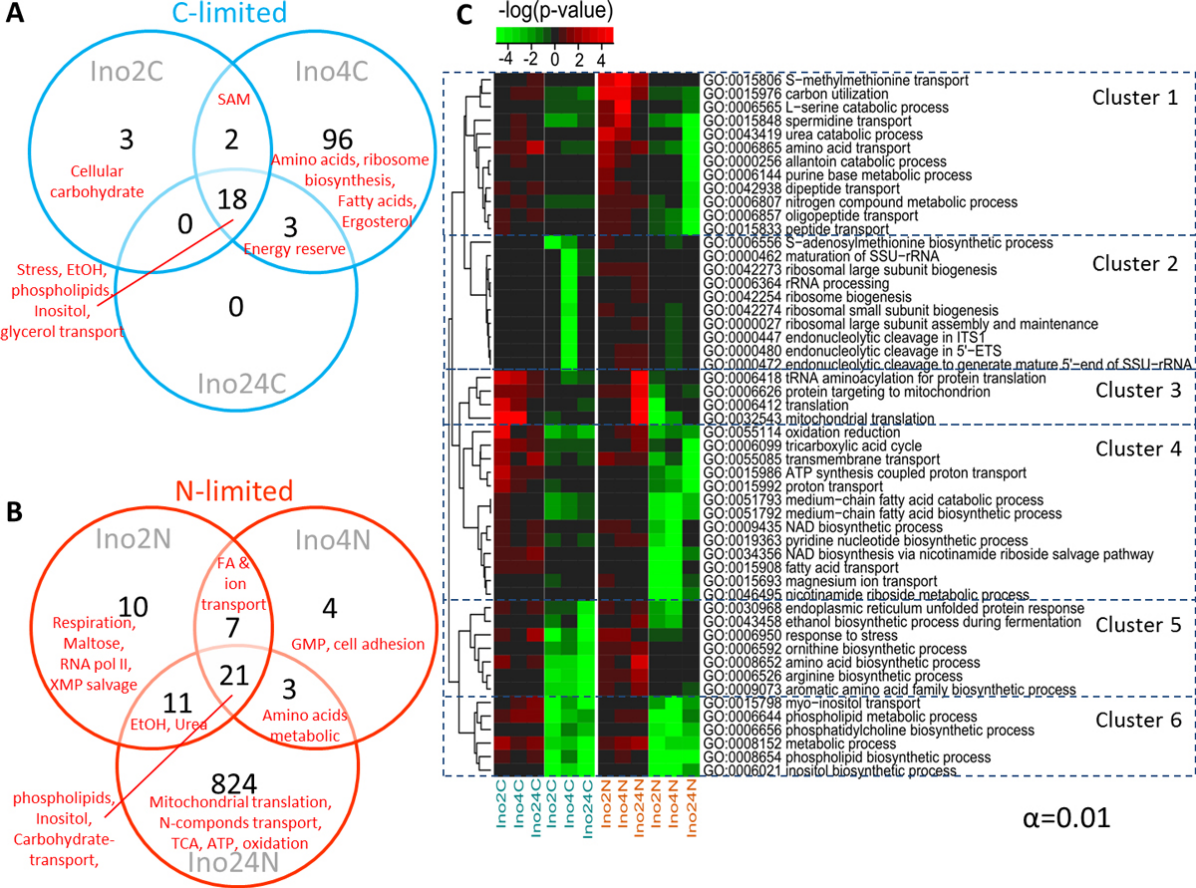
**Transcriptional data analysis of 3 different *INO*-deficient strain**. Transcriptional data analysis; Venn's diagram of significant genes of mutant strains caused by different deficient factors (single and double deletions) when focus on C-limitation (A) and N-limitation (B) separately, *p*-value < 0.01 were considered. (C) A heat map of overrepresented GO terms (Biological Process) of each factor comparison showing in range of -4 to 4 of log(*p*-value). The green color indicates down-regulation and red indicates up-regulation compared to the reference strain.

To identify specific transcriptional regulation of metabolism in response to deletion of *INO2 *and/or *INO4*, we performed transcriptome comparison of the three *INO *deficient mutants (defined as deficient factor in Figure 1C). For each strain we identified genes with significantly changed expression compared with the reference strain and then presented the results for the three strain as Venn diagrams for both C-limited (Figure 4A) and N-limited (Figure 4B) conditions. Even though it has been known that Ino2 and Ino4 form a heterodimeric transcription factor, we found some specific changes upon deletion of each of these genes (Figure 4A and 4C). Interestingly, *ino4*Δ has more significant genes due to the absent of *INO4 *compared with the two other mutants at C-limitation. At N-limitation, on the other hand, the effect is significantly higher when both *INO2 *and *INO4 *were deleted. Interestingly, most of the genes involving nitrogen compounds metabolism and carbon utilization (Figure 4C, Cluster 1) were down-regulated due to the absence of *INO2 *and *INO4 *but up-regulated when only *INO2 *or *INO4 *were knocked out individually at N-limitation. Moreover, we found that the genes involving ribosome biogenesis and rRNA processing (in cluster 2 Figure 4C) are dramatically down-regulated when *INO4 *was knocked out especially at C-limited conditions. The evidence showed that Ino4 (probably together with other TFs) could possibly play role in ribosome biogenesis (which supports protein synthesis). Surprisingly, most of the genes involving protein translation (Cluster 3 in Figure 4C, including mitochondria translation) were up-regulated in the double mutant at N-limited conditions while they were only up-regulated when either *INO2 *or *INO4 *were deleted at C-limitation. Consistently, the genes involving phospholipid, inositol, and fatty acids (UAS_INO_-contained genes in cluster 6, Figure 4C) were down regulated in all three strains at both C-limited and N-limited conditions. However, they were 2 other clusters that also captured down-regulated genes in all three *INO*-deleted strains, but different for the two nutrient limitations. The first group listed in cluster 4 (involving fatty acids biosynthesis and β-oxidation, TCA cycle, ATP, NAD, magnesium ion, and proton transport) were mainly down-regulated at N-limited condition. Cluster 5, on the other hand, contains genes involving ER-UPR and stress response, ethanol and amino acids biosynthesis that were mainly down-regulated only at C-limitation.

### Effects of *INO*-level on fatty acid biosynthesis

**Figure 5:**
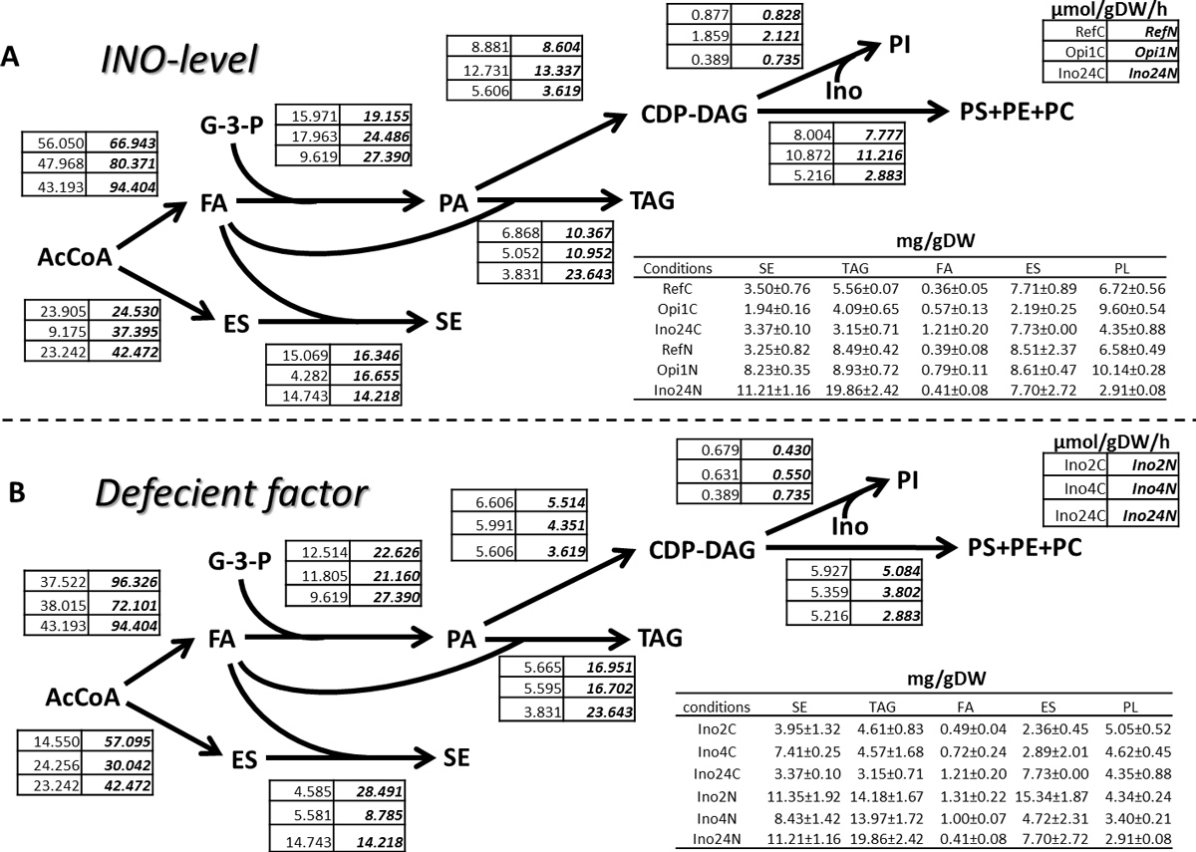
**Fluxes through the different reactions of the lipid biosynthetic pathways**. Fluxes through the different reactions of the lipid biosynthetic pathways for diferrent *INO*-levels (A) and diferrent deficient factors (B). All fluxes are shown in units of μmol/gDW/h. The normal font (left) and bold font (right) indicate the value from C-limitation and in N-limitation respectively. The level, in units of mg/gDW (±SD), of the different lipid species are shown in the table. (C = C-limited, N = N-limited).

With UASINO sites in their promoters, the expressions of fatty acid synthase genes such as *ACC1*, *FAS1*, and *FAS2 *are subject to control by the Ino2/Ino4 and Opi1 transcription factors [26,52,53]. At low *INO*-level strain, the fatty acid synthase genes (*FAS1 *and *FAS2*) were down-regulated at both C-limited and N-limited condition (Fig. S8 in additional file 1). However, single deletion of each *INO *gene seems to have effect only at C-limitation when *FAS1*, *FAS2*, and also *ACC1 *were down-regulated (Fig. S8 in additional file 1). In contrast, the high *INO*-level strains especially at N-limited condition showed an increase in *FAS2 *expression and none of the fatty acid synthase genes were down-regulated. This enhanced the ability to produce more FA needed for phospholipid biosynthesis. From the reporter metabolite heatmap (Fig. S2 in additional file 1), the FA pools and also fatty acyl-ACP were increasing for high *INO*-level strains especially at N-limited condition. All the low *INO*-level strains showed a dramatic decrease in FA and fatty acyl-ACP pools as reporter metabolites. However, it seems like the effects of lacking *INO *genes have higher effect at C-limited than at N-limited conditions (Fig. S2 in additional file 1). The transcription factors involving beta-oxidation, *OAF1 *and *PIP2*, were found as reporter TF which were highly up-regulated at high *INO*-level but down-regulated at low *INO*-level (Fig. S3 in additional file 1). Moreover, we found a significant increase in the free fatty acid (FA) pool of all the mutants compared to the reference strain (Figure 5). However, the low *INO*-level strains showed slightly higher amount of accumulated FAs due to the lower fluxes towards PLs. From the plots of correlation between each fatty acid chain and their elongase enzyme coding genes (Fig. S7 in additional file 1), we found a correlation between C18 fatty acid products (stearic and oleic acid) and the expression value of *ELO1 *to be about 0.65 while the correlation between C20 fatty acids and *ELO2*-*ELO3 *transcription were found in a negative direction (about -0.42 and -0.27, respectively). Presumably, C20 fatty acid might have a regulatory mechanism by repression their own elongase genes.

### Effects of *INO*-level on phospholipids biosynthesis and accumulation

As mentioned above the whole set of UAS_INO_-contained genes were extremely up-regulated at high *INO*-level (Fig. S8 in additional file 1). However, to get a clearer picture about the carbon channeling in lipid metabolism at the metabolite level, the fluxes in lipid biosynthesis (in units of μmol/gDW/h) were calculated from the measured lipid profiles at all conditions (Figure 5) and this provides a clear picture of the changes in the flux distribution in response to the different factors evaluated. These fluxes were affected by many factors at several levels, such as gene transcription, protein phosphorylation, and enzyme activity. At high *INO*-level, the fluxes through CDP-DAG and phospholipids synthesis pathway were higher than the reference about 45-55% at C-limitation and N-limitation, respectively. Moreover, the expression of the genes coding for myo-inositol transporter (*ITR1*) and choline transporter (*HNM1*) were highly up-regulated in *opi1*Δ. This supported the synthesis of PI and also enhanced the Kennedy pathway to produce more PC using choline and DAG as precursors and it was consistent with the highly up-regulation of the genes coding for Kenedy's pathway enzymes such as *CPT1 *and *CKI1 *(Fig. S8 in additional file 1). On the other hand, the *ITR1 *were constantly down-regulated in all low *INO*-level strains either double or single deletion in both limitation conditions. The low *INO*-level strain showed about 40-60% decreasing fluxes through the CDP-DAG pathway which caused a dramatically decrease in the phospholipid pool especially at N-limitation. The metabolite reporter heat map showed a large increase of PL pools in the high *INO*-level strain. In contrast, we found a dramatically decreasing of PLs in low *INO*-level compared to the reference strain.

Focusing on the deficient effects, double deletion of *INO *genes (especially at N-limitation) can cause more effect to the dramatically reduction of phospholipids than the effect of single deletions (Figure 5). About a 70% decrease in PLs can be found when both *INO *genes were deleted, but there were only 50% or 60% decreasing in PLs when *INO2 *or *INO4 *were deleted respectively. Even though this double deletion effects did not make much changes in the PLs pool at C-limitation, it still caused about 50% lower level of PI when compare to the single deletion strains. From the heatmap of reporter metabolites (Fig. S4 in additional file 1), S-adenosyl-L-methyonine or SAM (the only donor of PE methylation reaction) was strongly decreased in all low *INO*-level strains and this lead to a decrease of PLs (especially PC) since it is required for production of PC by Cho2 and Opi3.

### Effects of *INO*-level on storage lipids biosynthesis and accumulation

The production rate and accumulation of PC can cause ER stress and UPR activation which lead to the up-regulation of TAG and ES biosynthesis [54]. At N-limitation, the low *INO*-level strain (double mutant) could produce and accumulate TAG and SE about 1.2 folds more when compared to the high *INO*-level strain (*opi1*Δ) and about 1.5 folds when compare to the reference strain (Figure 5). Presumably, the down-regulation of *LPP1 *at both C- and N-limitation for the high *INO*-level strain led to a decrease in TAG biosynthesis and accumulation compared to the reference strain and other mutants (Fig. S8 in additional file 1). Consistently, almost the whole set of the ES genes (the green group in Fig. S8) were down-regulated compared to the reference and this was associated with a dramatically decreasing levels of ES and SE. This correlation between transcription of ES genes and ES level was also supported by the up-regulation of several ES genes in all low *INO*-level strains especially at N-limited condition (Fig. S8 in additional file 1). Interestingly, the double deletion strain showed a greater effect in increasing TAG only at N-limitation. This evidence was supported by the up-regulation of the *ARE1 *gene in the double deletion strain (Fig. S8 in additional file 1) which codes for the enzyme for the first step of SE biosynthesis. At C-limited condition, on the other hand, *ino2*Δ *ino4*Δ has a larger effect on decreasing the TAG level compared with the single deletion strains. Moreover, the fluxes through ES of the *ino2*Δ strain was about 80% lower when compare to *INO4 *deletion and double deletion at C-limited condition.

### Ino4 might have an extra function, beside the regulator of phospholipid biosynthesis, response to nitrogen starvation and amino acids starvations

It is possible that Ino4, but not Ino2, plays a role as a regulator for amino acid metabolism. It may not be Ino4 alone since Ino4, unlike Ino2, does not have a trans-activating domain (TAD) which is recognized for the RNA polymerase II complex [33,55,56]. In *ino4*Δ, the genes involving ribosome biogenesis and assembly were extremely down regulated especially at C-limited conditions and this was exactly the responsive process for amino acids (and of cause nitrogen) starvation. There are indications that the induction of Ino4-regulated lipid biosynthesis genes may be connected to the immediate need of membrane material used for the autophagocytosis process [57]. This process is utilized by yeast in order to regulate the equilibrium between proteins and the diminishing set of amino acids due to starvation conditions [58]. From the reporter metabolite heatmap (Fig. S4 in additional file 1), most of the amino acids were found to be greatly down-regulated (especially at N-limitation) in all low *INO*-level strains which pointed an amino acid starvation response.

To identify the extra function of Ino4 on protein biosynthesis, we also performed the Pearson correlation analysis of *INO4 *to all other genes based on the normalized expression values from transcriptome data of all strains and all conditions. Using the cut off at 0.50 absolute correlation value, about 470 genes were selected as the high correlated genes of *INO4 *(91 genes were positively correlated and 380 genes were negatively correlated). The overrepresentation of gene ontology categories (biological process) were identified and analyzed using BiNGO, a Cytoscape plugin [59]. From the *INO4 *positively correlated genes listed in table S2 in additional file 1 it confirmed the main function of Ino4 (together with Ino2 as listed in table S4 in additional file 1) as a positive regulators of lipid biosynthesis and inositol-choline transport involved genes as it has been reported before by many researchers. However, the list of *INO4 *negatively correlated genes in table S3 (please see additional file 1) showed the new findings that Ino4 is actually play an extra role as the negative regulator of the translation process, protein biosynthesis and assembly, and also involves in mitochondrial translation. Ino2, on the other hand, had about 200 negatively correlated genes that passed through the cut off at 0.50 absolute Pearson correlation value. This particular group contains many interesting genes involving protein transport, protein catabolic process, and proteolysis which might also responds for amino acid starvation as summarized in table S5 (please see in additional file 1).

### Linkage among sulfur-phospholipids, protein synthesis, and ER-UPR pathway

**Figure 6:**
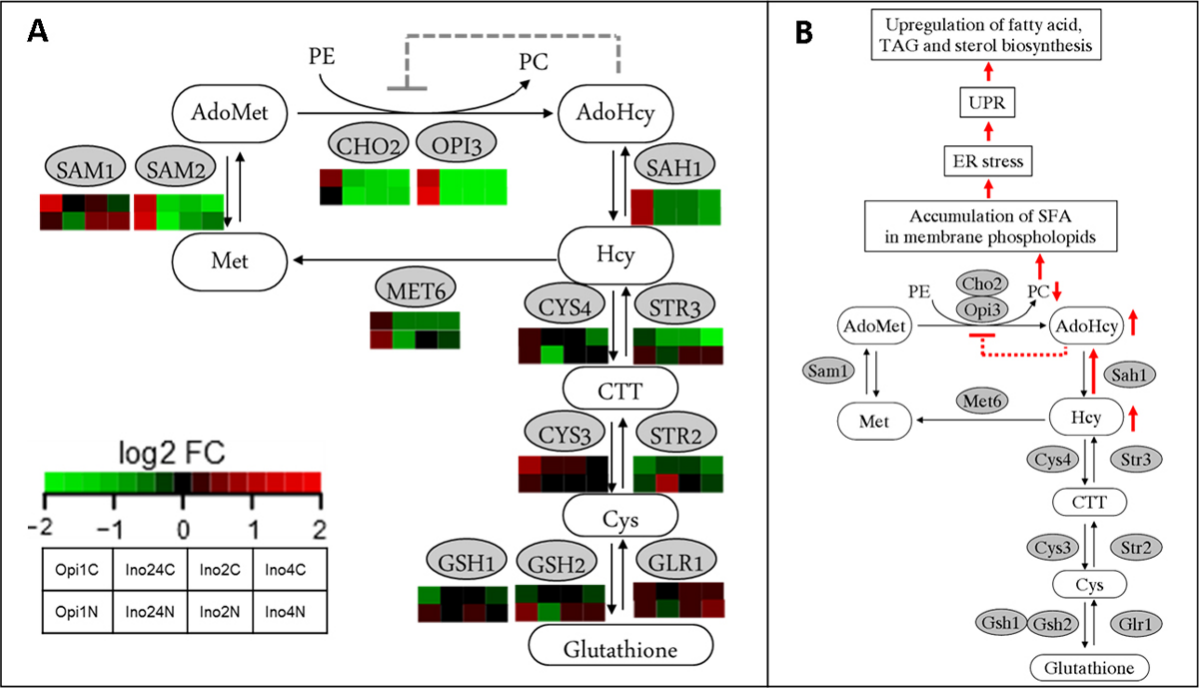
**The coupled-reaction of methylations of phosphoethanolamine from *S*-adenosyl-*L*-methionine (AdoMet) by Cho2 and Opi3 enzymes**. A comparison of expression level (log2 fold change) of each genes coding for sulfur-phospholipids coupled metabolism (A). The effects of low *INO*-level on ER stress and UPR inducing the up-regulation of FA and storage lipids (B).

**Figure 7:**
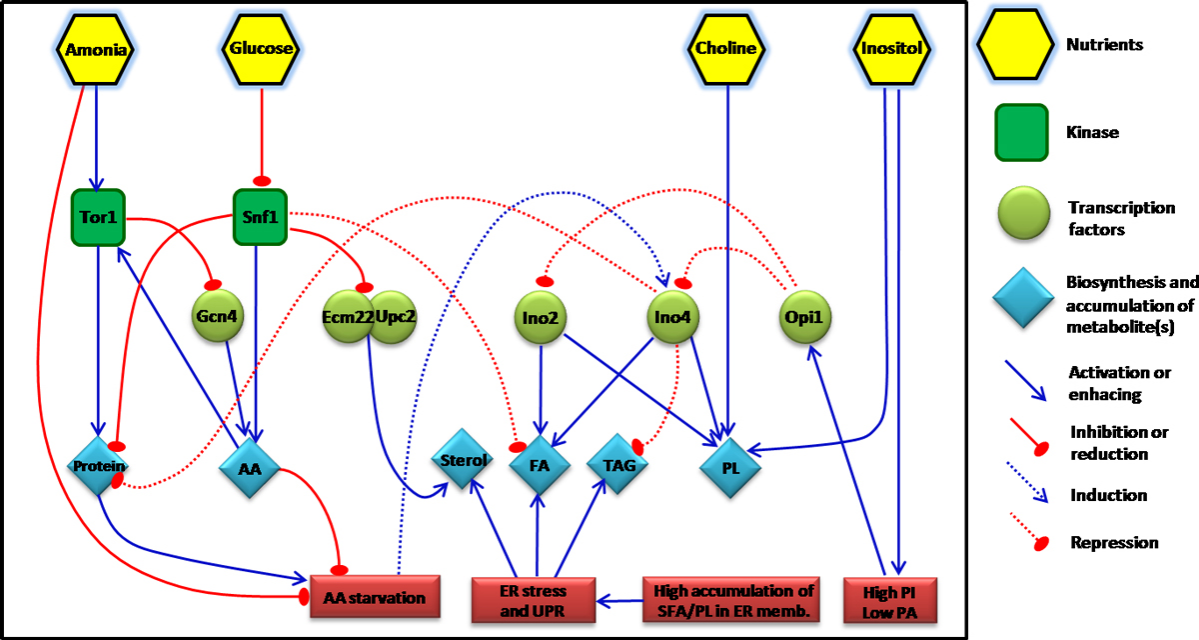
**Summary of the multilevel regulation network among amino acids biosynthesis, lipid metabolism, and ER-UPR**. The figure illustrate the regulatory model of yeast metabolism involving protein and lipid metabolism showing all the effects from nutrients-responsive mechanism to the kinase, gene expression, and metabolite level.

The double deletion of *INO2 *and *INO4 *at N-limitation showed some effects from amino acid starvation. UPR genes were also down-regulated when the synthesis level of amino acids was decreasing (less missed fold proteins). It also has been known that decreasing PC levels leads to the accumulation of saturated PC molecular species in the ER membrane which causes ER stress, UPR activation and these evidences lead to the up-regulation of FA, TAG, and sterol biosynthesis in the end [54]. Therefore, in the low *INO*-level strain, the *KAR2 *gene which is a responsive gene for ER-UPR was up-regulated especially at N-limitation. The high *INO*-level on the other hand, showed a large decrease in expression of *KAR2 *especially at C-limited condition. These evidences show that there is a linkage which plays a role in regulating the homeostasis among amino acids biosynthesis, phospholipids biosynthesis, and the ER-UPR pathway. As it has been reported before most of the genes coding for enzymes involved in cysteine-methionine biosynthesis, i.e. *MET2, MET8, MET14, MET16, SAH1, SAH2*, contain ICRE or UASINO sequences in their upstream regions [53], and these genes were up-regulated at high *INO*-level while *OPI1 *(the repressor) was disturbed. Consequently, we found down-regulation of *MET6 *and *SAH1 *and especially *SAH2 *in all low *INO*-level mutants, especially at C-limited condition. This leads to reduced synthesis and accumulation of storage lipids at C-limited condition compared to their references (Figure 6). This pointed out that amino acid synthesis genes might be controlled by the regulation of Snf1 kinase (via Gln3 or Gdh3) which is always activated at low glucose (C-limited) condition and consistent with our previous study [40]. Consistently, *MET6 *and *SAH1 *were up-regulated at high *INO*-level at N-limitations where Snf1 is consistently repressed by excess glucose in the culture media, and this leads to the slightly higher biosynthesis of TAG and hence TAG accumulation (Figure 6). Based on our findings and some supporting knowledge from the previous study [40], the overall regulation systems linking amino acids-protein synthesis, lipid metabolism and ER-UPR is summarised in Figure 7 which has kinase proteins and several transcription factors involved.

## Conclusions

Through integrated analysis of transcriptome and lipidome derived from robust experimental setup, it was possible to obtain a large-scale dataset that could be used to systematically identify correlations and associations between the different components. We were able to see the effect on expression of the genes involved in lipid biosynthesis and metabolic fluxes through lipid biosynthesis pathway. We found that there is an effect of Ino4, but not by Ino2, on the ribosome biogenesis and assembly which involves an amino acid starvation response. It points to an interesting link between lipid metabolism and the amino acid starvation response and we also found an effect of phospholipids on ER-UPR activation. Moreover, we found a close linkage among *INO *genes, amino acid genes, and probably Snf1 kinase in controlling lipid biosynthesis and accumulation. Following our analysis by genome-wide strategy and analysis of generated complex data by integrated analysis approach enable us to explore correlations and association of changes in a concerted fashion.

## Competing interests

The authors declare that they have no competing interests.

## Authors' contributions

PC conducted all the experiments, analyzed the data and prepared the manuscript. IN assisted with the data analysis for transcriptome and lipid analysis. JN edited and approved manuscript.

## Supplementary Material

Additional file 1 Supplementary Information to "Integrated analysis of the transcriptome-lipidome reveals the effects of *INO*-level (*INO2 *and *INO4*) on lipid metabolism in yeast" Click here for file

## Declarations

Part of this work was financed by Chalmers Foundation, the Knut and Alice Wallenberg Foundation and the Swedish Research Council (Vetenskapsrådet). We also acknowledge funding from the EU-funded project UNICELLSYS.

This article has been published as part of *BMC Systems Biology *Volume 7 Supplement 2, 2013: Twelfth International Conference on Bioinformatics (InCoB2013): Bioinformatics. The full contents of the supplement are available online at http://www.biomedcentral.com/bmcsystbiol/supplements/7/S3.
