# Supplementary material for: Integrated analysis, transcriptome-lipidome, reveals the effects of INO-level (INO2 and INO4) on lipid metabolism in yeast
Source: BMC Syst Biol. 2013 Oct 16;7(Suppl 3):S7. doi: 10.1186/1752-0509-7-S3-S7 (PMC3852131; doi:10.1186/1752-0509-7-S3-S7)
Supplement: Additional file 1 — Supplementary Information to "Integrated analysis of the transcriptome-lipidome reveals the effects of INO-level (INO2 and INO4) on lipid metabolism in yeast" [file 1752-0509-7-S3-S7-S1.pdf]

## Supplementary Information to

### Title: Integrated analysis of the transcriptome-lipidome reveals the effects of *INO*-level (*INO2* and *INO4*) on lipid metabolism in yeast

---

**Pramote Chumnanpuen**[1,2], **Intawat Nookaew**[2], **and Jens Nielsen**[2]*

[1] Department of Zoology, Faculty of Science, Kasetsart University, Bangkok, Thailand

[2] Systems and Synthetic Biology, Department of Chemical and Biological Engineering, Chalmers University of Technology, Gothenburg, Sweden

*Corresponding Author:

Jens Nielsen

Email: nielsenj@chalmers.se

Tel: +46 31 772 38 04

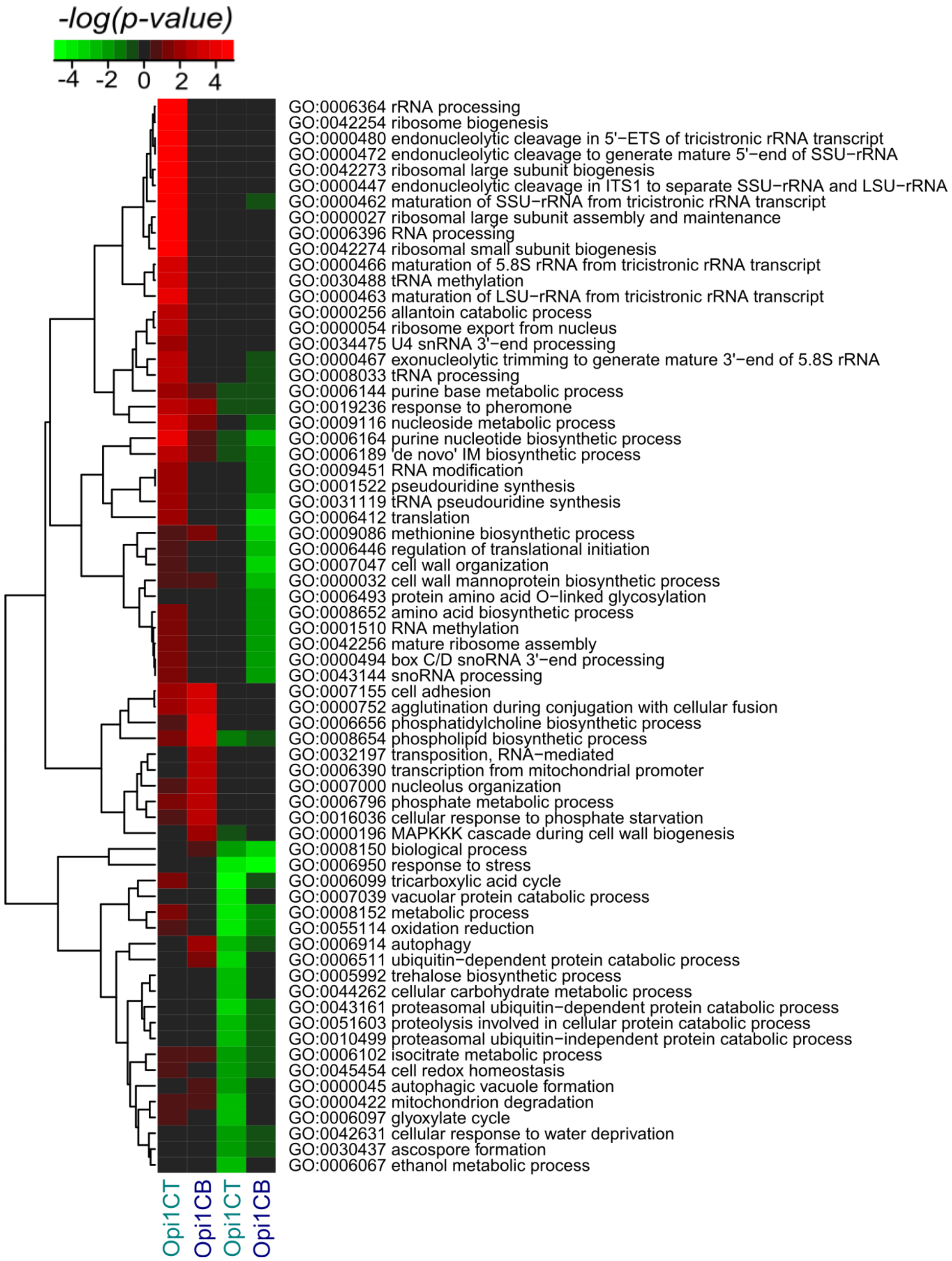


**Figure S1.** A heat map of over-representation of GO terms (Biological Process) responded to the 2 different phases of *opi1*Δ at C-limitation (top phase and bottom phase of $CO_2$ profile)

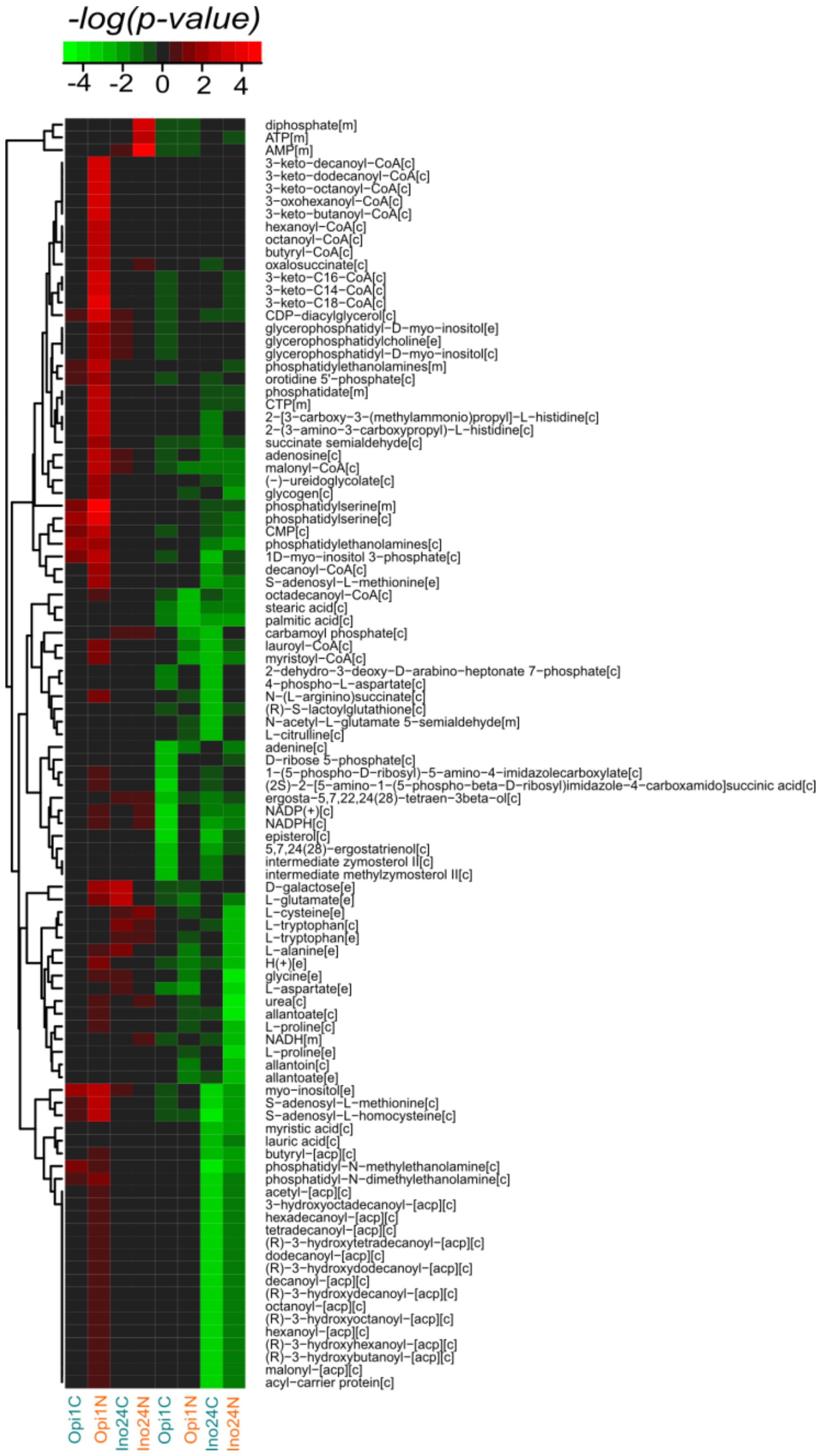


**Figure S2.** A heat map of reporter Metabolites of *INO*-level comparison

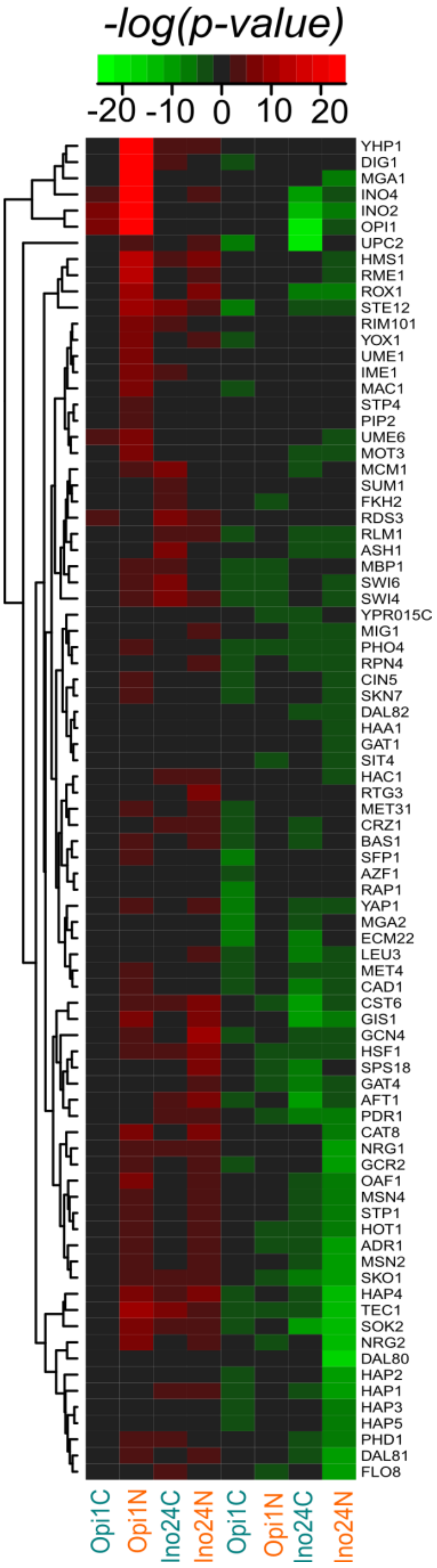


**Figure S3.** A heat map of reporter transcription factors of *INO*-level comparison

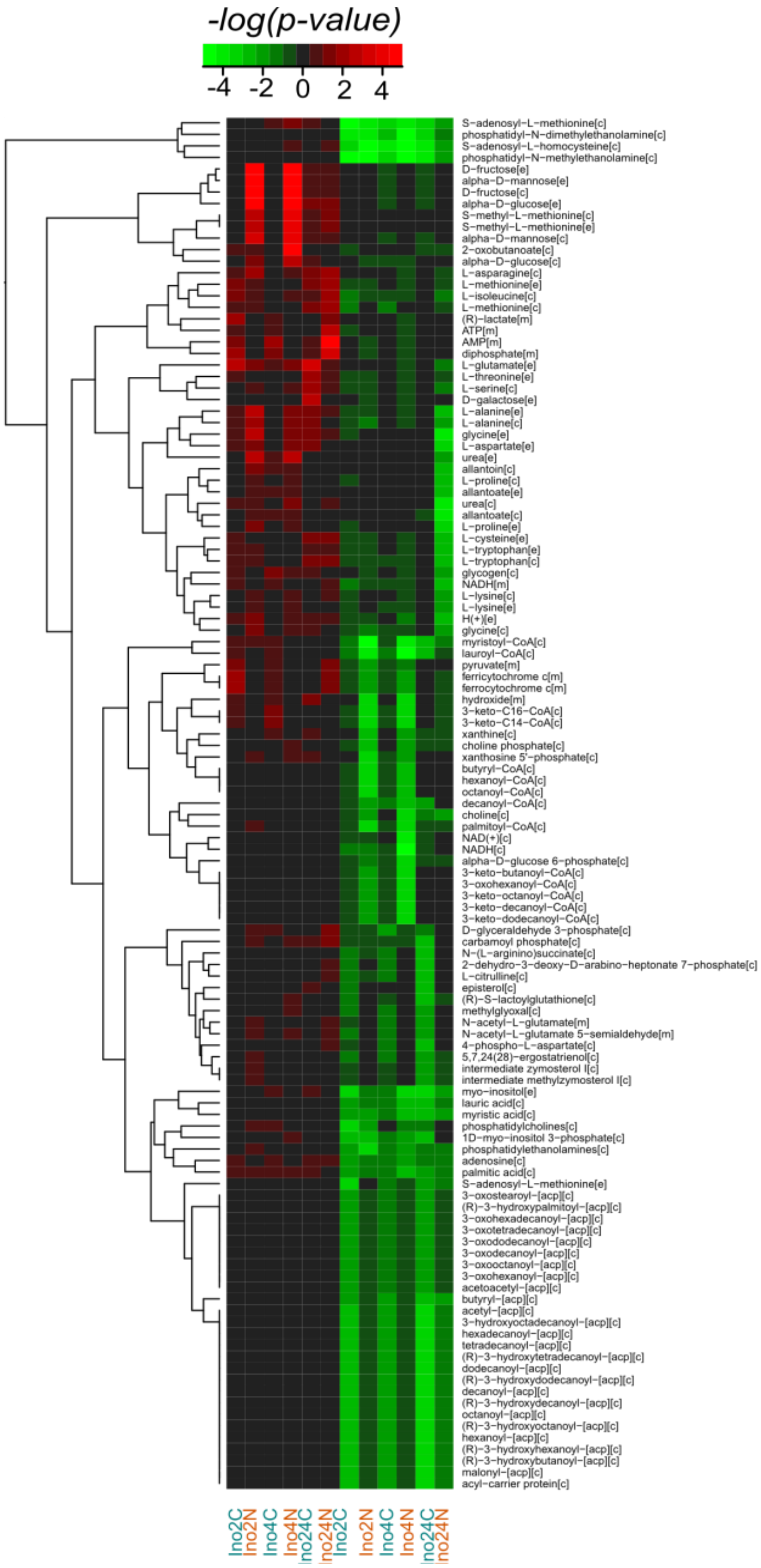


**Figure S4.** A heat map of reporter metabolites of deficient effects comparison

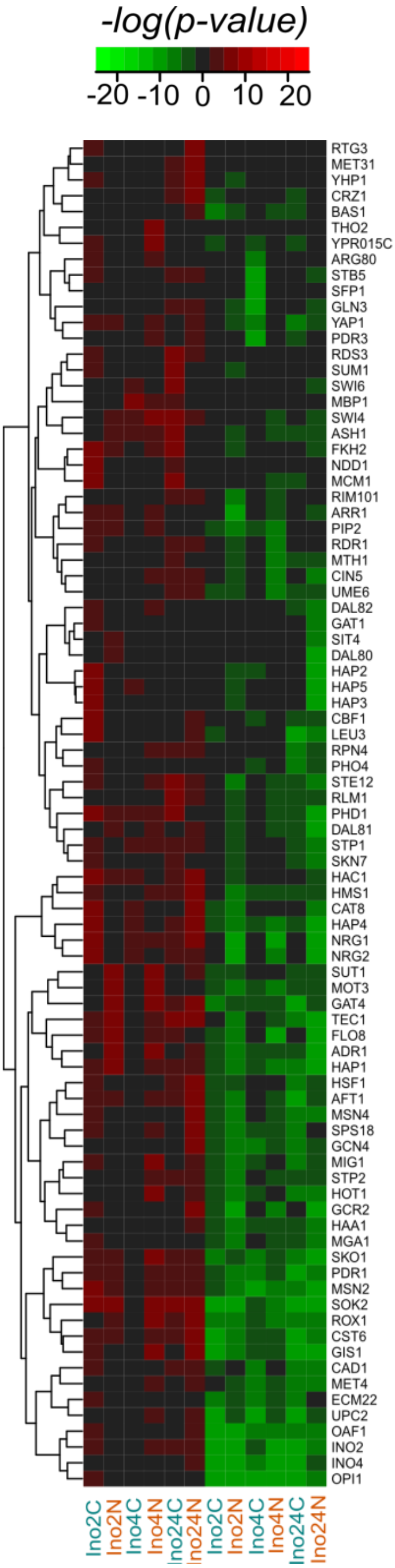


**Figure S5.** A heat map of reporter transcription factors of deficient effects comparison.

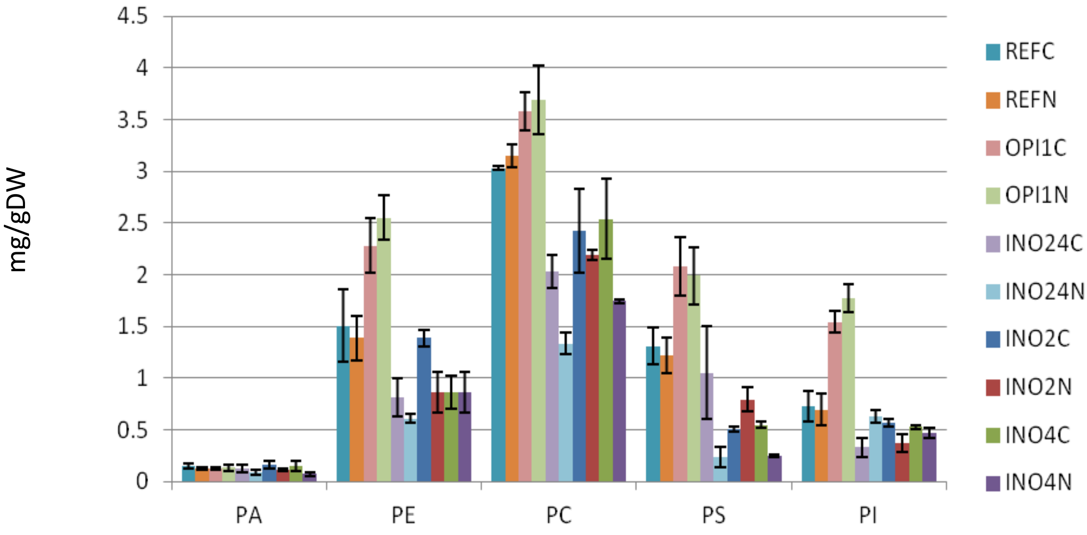


**Figure S6.** Abundance of different phospholipid species in units of mg/gDW

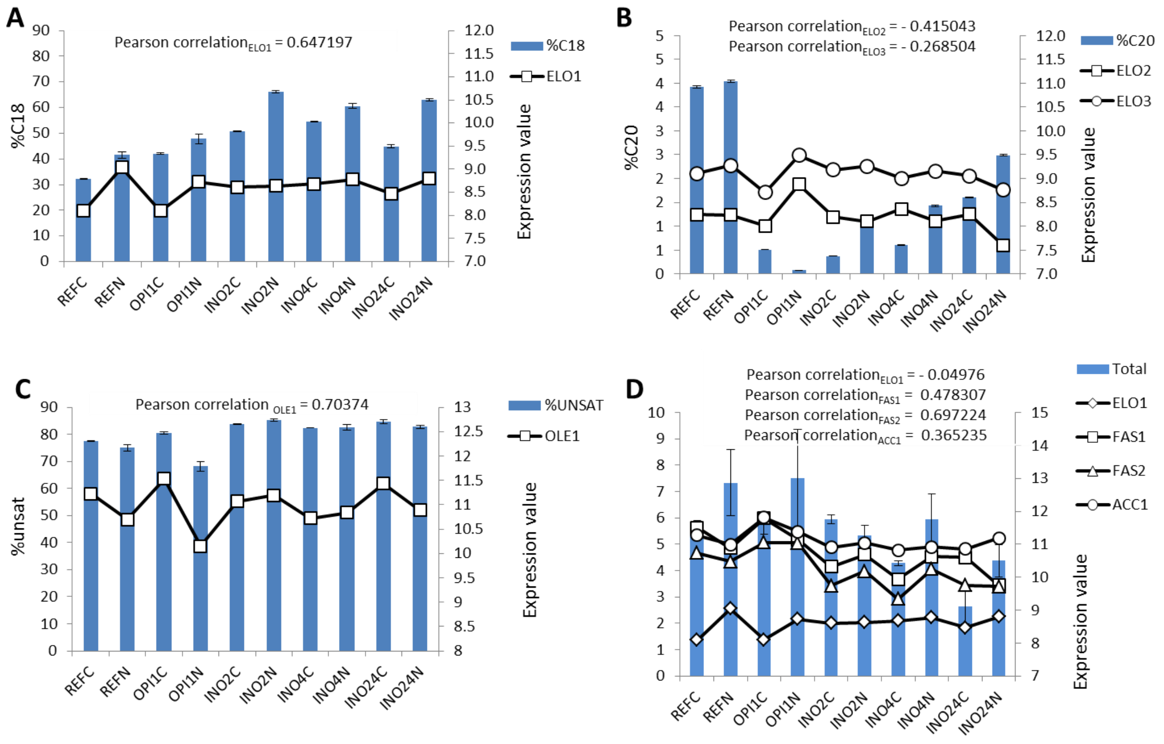


**Figure S7.** Correlations between the transcription level of fatty acid enzymes (synthase and elongases)and their products. (**A**) Correlation between expression value (log2) of *ELO1* and percentage of C18 fatty acids. (**B**) Correlation between expression value (log2) of *ELO2, ELO3* and percentage of C20 fatty acids. (**C**) Correlation between expression value (log2) of *OLE1* and percentage of unsaturated fatty acids. (**D**) Correlation between expression value (log2) of *ELO1, FAS1, FAS2, ACC1* and total fatty acids in the unit of mmol/gDW.

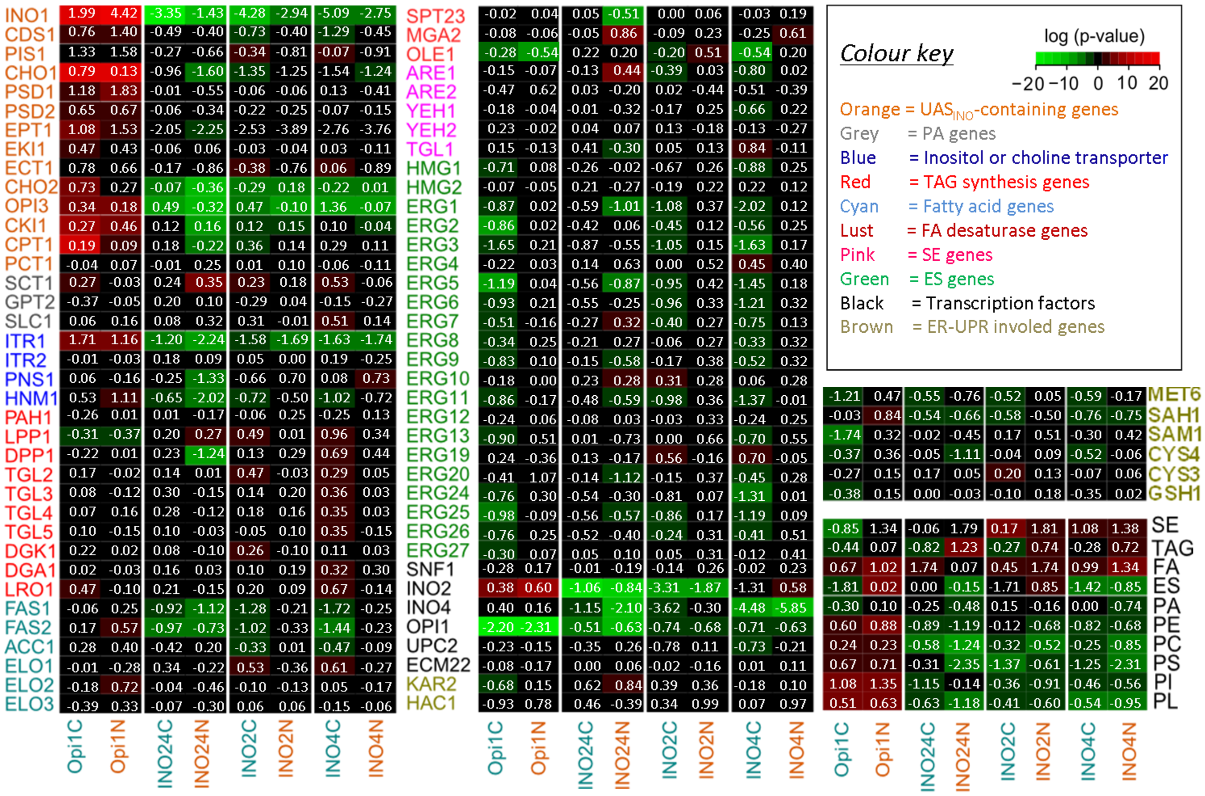

**Figure S8.** The heatmap of overrepresented lipid involved genes and all lipid classes showing in the range of -20 to 20 of log (*p*-value) from T-test analysis. The green color indicates down-regulation and red indicates up-regulation compared to the reference strain.

**Table S1.** The list of average molecular weights for phospholipid amount calculation.

| Lipids | Average MW (g/mol) |
|---|---|
| PA | 685.10 |
| PE | 706.19 |
| PC | 748.27 |
| PS | 772.18 |
| PI | 842.29 |

**Table S2**. Summary of over-represented biological processes in significant genes which are positively correlated to *INO4* showing higher than 0.50 in Pearson correlation values.

| Description | p-value | Gene in the test |
|---|---|---|
| cellular process | 1.04E-13 | APP1, ABC1, MDJ1, UBP10, SAP4, BNA5, CRH1, NSP1, EPT1, RET2, TRK2, GLT1, EMP47, PEX30, RTA1, RGT2, LCB5, SPO71, PXR1, AAD15, SPO74, HIR3, MAM1, HFM1, DLD1, SRC1, ALR2, MGR1, PAN3, TOM70, VPS45, NRK1, PIM1, UBR2, SLN1, BEM4, FAR11, IMH1, RAD2, THI7, NUP42, MPE1, SRO77, YTA7, ACT1, PCL9, ECM18, CCC2, PYC1, GAT1, VBA2, GAT3, DUG3, RIC1, RPT6, JEM1, ARG4, COY1, AQR1, IZH1, HTD2, SMA1, RPS19A, SMP3, GDE1, ECM5, YIP3, RNT1, SSA2, HNM1, RPT4, ARF2, UBA1, SAM50, RIT1, PHO5, ADD66, MSD1, PTC2, SPR28, MSR1, HEM2, MLH2, SAL1, MF(ALPHA)1, SKS1, KAR2, PBA1, FRE2, CUP1-2, CUP1-1, MPH1, GUP1, HST3, RNR3, MCD4, NMA2, VAM6, ADO1, HSP60, TSA1, YRM1, UFD2, CDS1, SPO23, NOC2, RAD50, FAS1, FAS2, EEB1, MSB2, NOT3, CTM1, MOT2, SSC1, BOI1, MOT3, HUG1, THI3, YME2, DAK1, MSP1, ATG11, FHL1, INO1, APC1, INO2, SPP382, AFG3, RPI1, BET3, NTA1, SPO11, BET4, CKI1, SPO13, CHO1, KTI11, AFG1, TYE7, MAS1, CHO2, THI21, PET112, PEX10, YRF1-5, CWC25, YRF1-6, YRF1-7, AAD3, YRF1-1, PMT3, YRF1-2, YRF1-3, YRF1-4, STV1, SAM3, SAM2, SRS2, GPI17, CBP2, MOG1, COG4, APS1, BCK1, HSP78, AAD4, ABZ1, INO80, VMR1, BSC6, OPI1, QDR1, OPI3, YDC1, TOP2, RPN9, ATG5, CLP1, HXT11, HXT12, PDI1, RPN6, FZO1, REC104, ITR1, PSD1, XDJ1, RGM1, ENT5, MMR1, URK1, SEC39, PMR1, RAD14, NPL4, BCH1, SMF1, CHS5, UBX2, SEC21, BDS1, TAT1, FKS3, SEC24, MET6, FAA1, SEC27, UBX6, MUC1, URA8, NIS1, BPH1, SET2, DNA2, SAC6, SCL1, NNT1, AAH1, ZIP1, CPT1, RSF1, ERO1, ALG1, YJU2, PEX6, TPO4, BAP3, SIC1, RSC30, JLP1, AGA1, PCP1, RPS23A, SEY1, VPS16, RPS23B, UGO1, MGM1, NRG2, MSK1, CYS4, RSE1, REV1, SIT4, LHS1, IMD2, RPL31A, DRE2, GPB1, COQ1, SCJ1, LYS21, HSC82, BFR2, DSE4, DSE3, LYP1, SPT6, MNN5, HAT2, SDA1, KRE5, SEC61, DSS1, RPA34, MATALPHA1, DNF1, DIT2, MEP3, DIT1, ICT1, MSI1, PDR1, NAM9, VPS35, ZWF1, NAM2, LPP1, PDR3, SCO1, MDL1, FOL1, SSM4, JIP5, ACC1, MSH5, SSK22, SAH1, EHT1, MON1, PHR1, PRM7, RPN11, YTA12, FIG2, CRN1, NEW1, RPB10, PHB2, TDP1, VPS27, HMLALPHA1, UTR2 |
| phospholipid biosynthetic process | 3.72E-08 | URA8, CPT1, SMP3, MCD4, GAT1, INO1, INO2, PSD1, EPT1, CDS1, OPI1, CKI1, OPI3, GPI17, CHO1, ICT1, GUP1, CHO2 |
| establishment of localization | 8.45E-08 | NSP1, VMR1, BSC6, OPI1, QDR1, TRK2, RET2, EMP47, PDR11, ATG5, RGT2, HXT11, HXT12, SRC1, ALR2, TOM70, VPS45, ITR1, IMH1, ENT5, NUP42, THI7, SRO77, ACT1, SEC39, PMR1, NPL4, CCC2, BCH1, VBA2, SMF1, RIC1, CHS5, UBX2, COY1, AQR1, SEC21, TAT1, SEC24, FAA1, RPS19A, UBX6, SEC27, NIS1, BPH1, YIP3, SSA2, HNM1, ARF2, SAC6, SAM50, AUS1, ERO1, TPO4, PEX6, SAL1, BAP3, SKS1, KAR2, PCP1, FRE2, GUP1, VPS16, UGO1, MSK1, MCD4, VAM6, LHS1, YRM1, HSP60, NOC2, SCJ1, BFR2, SSC1, LYP1, ATG11, MSP1, AFG3, BET3, BET4, SDA1, SEC61, DSS1, MEP3, DNF1, AFG1, MAS1, SYG1, VPS35, PEX10, PDR5, SCO1, MDL1, ACC1, STV1, SAM3, MON1, MOG1, SFH5, COG4, APS1, YTA12, NEW1, HSP78, VPS27 |
| transport | 9.87E-08 | NSP1, VMR1, BSC6, QDR1, TRK2, RET2, EMP47, PDR11, ATG5, RGT2, HXT11, HXT12, ALR2, TOM70, VPS45, ITR1, IMH1, ENT5, NUP42, THI7, SRO77, ACT1, SEC39, PMR1, NPL4, CCC2, BCH1, VBA2, SMF1, RIC1, CHS5, UBX2, COY1, AQR1, SEC21, TAT1, SEC24, FAA1, RPS19A, SEC27, NIS1, BPH1, YIP3, SSA2, HNM1, ARF2, SAC6, SAM50, AUS1, ERO1, TPO4, PEX6, SAL1, BAP3, SKS1, KAR2, PCP1, FRE2, GUP1, VPS16, UGO1, MSK1, MCD4, VAM6, LHS1, YRM1, HSP60, NOC2, SCJ1, BFR2, SSC1, LYP1, ATG11, MSP1, AFG3, BET3, BET4, SDA1, SEC61, DSS1, MEP3, DNF1, AFG1, MAS1, SYG1, VPS35, PEX10, PDR5, MDL1, SCO1, ACC1, STV1, SAM3, MON1, MOG1, SFH5, COG4, APS1, YTA12, NEW1, HSP78, VPS27 |

| | | |
|---|---|---|
| localization | 1.34E-07 | NSP1, VMR1, BSC6, OPI1, QDR1, TRK2, RET2, EMP47, PDR11, ATG5, RGT2, HXT11, HXT12, SRC1, ALR2, TOM70, VPS45, ITR1, IMH1, ENT5, NUP42, THI7, SRO77, YTA7, MMR1, ACT1, SEC39, PMR1, NPL4, CCC2, BCH1, VBA2, SMF1, RIC1, CHS5, UBX2, COY1, AQR1, SEC21, TAT1, SEC24, FAA1, RPS19A, UBX6, SEC27, NIS1, BPH1, YIP3, SSA2, HNM1, ARF2, SAC6, SAM50, AUS1, ERO1, TPO4, PEX6, SAL1, BAP3, SKS1, KAR2, PCP1, FRE2, GUP1, VPS16, UGO1, MSK1, MCD4, VAM6, LHS1, YRM1, HSP60, NOC2, SCJ1, BFR2, SSC1, LYP1, ATG11, MSP1, AFG3, BET3, BET4, SDA1, SEC61, SPO13, DSS1, DNF1, MEP3, AFG1, MAS1, SYG1, VPS35, PDR5, PEX10, SCO1, MDL1, ACC1, STV1, SAM3, MON1, MOG1, SFH5, COG4, APS1, YTA12, CRN1, NEW1, HSP78, PHB2, VPS27 |
| cellular lipid metabolic process | 2.55E-07 | CPT1, ALG1, GAT1, INO1, INO2, EPT1, OPI1, CKI1, OPI3, YDC1, CHO1, ICT1, LCB5, IZH1, HTD2, GUP1, CHO2, FAA1, LPP1, URA8, HST3, SMP3, ACC1, GDE1, MCD4, EHT1, PSD1, CDS1, GPI17, FAS1, FAS2, EEB1, COQ1 |
| establishment of protein localization | 3.39E-07 | NSP1, PEX6, OPI1, RET2, ATG5, KAR2, PCP1, VPS16, SRC1, TOM70, VPS45, VAM6, LHS1, HSP60, IMH1, ENT5, NUP42, SCJ1, ACT1, SEC39, BFR2, NPL4, SSC1, BCH1, ATG11, MSP1, AFG3, BET4, SDA1, SEC61, RIC1, CHS5, UBX2, DNF1, AFG1, MAS1, SEC21, VPS35, SEC24, PEX10, UBX6, SEC27, ACC1, STV1, BPH1, YIP3, MON1, SSA2, MOG1, COG4, APS1, YTA12, ARF2, HSP78, SAM50, VPS27 |
| intracellular protein transport | 7.93E-07 | MSP1, NSP1, ATG11, PEX6, AFG3, BET4, SEC61, RIC1, RET2, DNF1, ATG5, PCP1, KAR2, AFG1, MAS1, SEC21, VPS35, VPS16, SEC24, PEX10, SEC27, ACC1, TOM70, VPS45, LHS1, BPH1, HSP60, MON1, SSA2, IMH1, MOG1, NUP42, APS1, YTA12, HSP78, SAM50, VPS27, SSC1 |
| lipid biosynthetic process | 1.28E-06 | URA8, CPT1, SMP3, ACC1, MCD4, GAT1, INO1, INO2, PSD1, EPT1, OPI1, CDS1, CKI1, OPI3, GPI17, YDC1, FAS1, FAS2, COQ1, CHO1, ICT1, HTD2, CHO2, GUP1 |
| glycerophospholipid biosynthetic process | 1.57E-06 | CPT1, SMP3, MCD4, PSD1, EPT1, CDS1, CKI1, GPI17, OPI3, CHO1, ICT1, GUP1, CHO2 |
| macromolecule localization | 1.66E-06 | NSP1, PEX6, OPI1, RET2, PDR11, ATG5, PCP1, KAR2, VPS16, MSK1, SRC1, TOM70, VPS45, LHS1, VAM6, HSP60, IMH1, ENT5, NUP42, YTA7, SCJ1, ACT1, SEC39, BFR2, NPL4, SSC1, BCH1, ATG11, MSP1, AFG3, SDA1, BET4, SEC61, SPO13, RIC1, CHS5, DSS1, UBX2, DNF1, AFG1, MAS1, SEC21, VPS35, SEC24, PEX10, FAA1, RPS19A, UBX6, SEC27, ACC1, STV1, BPH1, YIP3, MON1, SSA2, MOG1, SFH5, COG4, APS1, YTA12, NEW1, ARF2, HSP78, SAM50, VPS27, AUS1 |
| protein transport | 1.99E-06 | NSP1, PEX6, RET2, ATG5, KAR2, PCP1, VPS16, TOM70, VPS45, VAM6, LHS1, HSP60, IMH1, ENT5, NUP42, SCJ1, ACT1, SEC39, BFR2, NPL4, SSC1, BCH1, ATG11, MSP1, AFG3, BET4, SDA1, SEC61, RIC1, CHS5, UBX2, DNF1, AFG1, MAS1, SEC21, VPS35, SEC24, PEX10, SEC27, ACC1, BPH1, SSA2, MON1, MOG1, COG4, APS1, YTA12, ARF2, HSP78, SAM50, VPS27 |
| phospholipid metabolic process | 2.48E-06 | URA8, CPT1, SMP3, GDE1, MCD4, GAT1, INO1, INO2, PSD1, EPT1, OPI1, CDS1, CKI1, OPI3, GPI17, CHO1, ICT1, GUP1, CHO2, LPP1 |
| glycerolipid biosynthetic process | 2.50E-06 | CPT1, SMP3, MCD4, PSD1, EPT1, CDS1, CKI1, GPI17, OPI3, CHO1, ICT1, GUP1, CHO2 |
| protein localization | 3.16E-06 | NSP1, PEX6, OPI1, RET2, ATG5, KAR2, PCP1, VPS16, SRC1, TOM70, VPS45, VAM6, LHS1, HSP60, IMH1, ENT5, NUP42, YTA7, SCJ1, ACT1, SEC39, BFR2, NPL4, SSC1, BCH1, ATG11, MSP1, AFG3, SDA1, BET4, SEC61, SPO13, RIC1, CHS5, UBX2, DNF1, AFG1, MAS1, SEC21, VPS35, SEC24, PEX10, UBX6, SEC27, ACC1, STV1, BPH1, YIP3, MON1, SSA2, MOG1, COG4, APS1, YTA12, ARF2, HSP78, SAM50, VPS27 |
| phosphatidylcholine biosynthetic process | 3.28E-06 | CPT1, PSD1, CHO2, EPT1, CKI1, OPI3 |
| telomere maintenance via recombination | 3.52E-06 | YRF1-5, YRF1-6, YRF1-7, YRF1-1, YRF1-2, YRF1-3, YRF1-4, RAD50 |
| organophosphate metabolic process | 4.22E-06 | URA8, CPT1, SMP3, GDE1, MCD4, GAT1, INO1, INO2, PSD1, EPT1, OPI1, CDS1, CKI1, OPI3, GPI17, CHO1, ICT1, GUP1, CHO2, LPP1 |

| | | |
|---|---|---|
| intracellular transport | 5.50E-06 | NSP1, PEX6, SAL1, RET2, EMP47, ATG5, KAR2, PCP1, VPS16, MSK1, TOM70, VPS45, LHS1, HSP60, NOC2, IMH1, ENT5, NUP42, SRO77, ACT1, SEC39, SSC1, BCH1, ATG11, MSP1, AFG3, BET3, BET4, SDA1, SEC61, RIC1, CHS5, DSS1, DNF1, COY1, AFG1, MAS1, SEC21, VPS35, SEC24, PEX10, RPS19A, SEC27, ACC1, BPH1, YIP3, MON1, SSA2, MOG1, COG4, APS1, YTA12, NEW1, ARF2, HSP78, SAM50, VPS27 |
| cellular protein localization | 6.03E-06 | NSP1, MSP1, ATG11, PEX6, AFG3, BET4, SEC61, SPO13, RIC1, RET2, DNF1, ATG5, PCP1, KAR2, AFG1, SEC21, MAS1, VPS35, VPS16, SEC24, PEX10, SEC27, ACC1, TOM70, VPS45, LHS1, BPH1, HSP60, MON1, SSA2, IMH1, MOG1, NUP42, APS1, YTA12, YTA7, HSP78, SAM50, VPS27, SSC1 |
| intracellular protein transmembrane transport | 8.00E-06 | YTA12, TOM70, HSP78, PCP1, KAR2, LHS1, AFG3, HSP60, SEC61, SSA2, SSC1 |
| lipid metabolic process | 8.77E-06 | CPT1, ALG1, GAT1, INO1, INO2, EPT1, OPI1, CKI1, OPI3, YDC1, CHO1, ICT1, LCB5, IZH1, HTD2, GUP1, CHO2, FAA1, LPP1, URA8, HST3, SMP3, ACC1, GDE1, MCD4, EHT1, PSD1, CDS1, GPI17, FAS1, FAS2, EEB1, COQ1 |
| transmembrane transport | 1.08E-05 | NSP1, VBA2, TPO4, AFG3, VMR1, BAP3, SAL1, BSC6, SEC61, QDR1, TRK2, MEP3, RGT2, PCP1, KAR2, AQR1, FRE2, TAT1, HXT11, HXT12, UGO1, MDL1, ALR2, TOM70, LHS1, STV1, ITR1, HSP60, YRM1, SAM3, SSA2, HNM1, NUP42, THI7, YTA12, HSP78, NPL4, CCC2, SSC1, LYP1 |
| response to stimulus | 1.12E-05 | MDJ1, MLH2, INO80, OPI1, MF(ALPHA)1, QDR1, SKS1, RSC30, ATG5, RTA1, AGA1, RGT2, KAR2, LCB5, FRE2, CUP1-2, CUP1-1, AAD15, MPH1, SEY1, SLH1, PAU1, REV1, PAN3, DAN4, SIT4, LHS1, HSP60, TSA1, YRM1, SLN1, UFD2, BEM4, FAR11, IMH1, RAD50, GPB1, RAD2, SCJ1, MSB2, HSC82, ACT1, RAD14, SSC1, HUG1, SPT6, DAK1, GAT1, HAT2, INO2, ARR2, ICT1, AQR1, AFG1, MSI1, IZH1, PDR1, BDS1, PDR5, ZWF1, PDR3, SCO1, AAD3, MSH5, SSK22, BPH1, SSA2, SRS2, PHR1, DNA2, FIG2, BCK1, ARF2, HSP78, TDP1, SAC6, PHO5, AAD4, PTC2 |
| phosphatidylcholine metabolic process | 1.32E-05 | CPT1, PSD1, CHO2, EPT1, CKI1, OPI3 |
| cellular macromolecule localization | 1.39E-05 | NSP1, MSP1, ATG11, PEX6, AFG3, BET4, SEC61, SPO13, RIC1, RET2, DNF1, ATG5, PCP1, KAR2, AFG1, SEC21, MAS1, VPS35, VPS16, SEC24, PEX10, SEC27, ACC1, TOM70, VPS45, LHS1, BPH1, HSP60, MON1, SSA2, IMH1, MOG1, NUP42, APS1, YTA12, YTA7, HSP78, SAM50, VPS27, SSC1 |
| establishment of localization in cell | 1.41E-05 | NSP1, PEX6, SAL1, RET2, EMP47, ATG5, KAR2, PCP1, VPS16, MSK1, TOM70, VPS45, LHS1, HSP60, NOC2, IMH1, ENT5, NUP42, SRO77, ACT1, SEC39, PMR1, SSC1, BCH1, ATG11, MSP1, AFG3, BET3, SDA1, BET4, SEC61, RIC1, CHS5, DSS1, UBX2, DNF1, COY1, AFG1, MAS1, SEC21, VPS35, SEC24, PEX10, RPS19A, SEC27, ACC1, BPH1, YIP3, MON1, SSA2, MOG1, COG4, APS1, YTA12, NEW1, ARF2, HSP78, SAM50, VPS27 |

**Table S3**. Summary of over-representative biological process in of the significant genes which are negatively correlated to *INO4* showing lower than -0.50 in Pearson correlation values.

| Description | p-value | Gene in the test |
|---|---|---|
| mitochondrion organization | 1.60E-68 | MRPL40, YML6, MRPS35, MDJ1, MRS11, MRP51, MRPL36, MRPL37, MRPL38, MRPL32, MGR2, FZO1, MRPL50, TOM70, MHR1, MSW1, PAM17, SLM5, MRPL49, ATP12, ATP11, MSF1, MRPS18, MST1, TOM20, DIA4, MDM34, QRI7, SHE9, MSE1, MDM35, SWS2, MRPS28, PNT1, TIM13, CCE1, SAM50, MSD1, MSR1, RPO41, MRP13, RRF1, SAM37, PCP1, MRP17, UGO1, COX18, MGM1, MSK1, MRPL1, MSS2, COX23, MRPL6, ACO1, MRPL8, MRPL7, SHY1, MRPS5, HSP60, MRP4, MRPS9, RSM7, MRP7, MRPS8, PET123, BCS1, MRP2, SSC1, YME2, RSM18, RSM19, MSP1, MIA40, AFG3, TPM1, ISM1, MSY1, MRPL20, IMG1, MRPL11, IMG2, MRPL10, MRPL13, MRPL15, MRPL17, MRPL16, MAS1, MGE1, MRPL19, NAM9, RSM10, MRF1, PET112, NAM2, ERV1, RSM26, TCM62, MRP20, MRP21, CBP4, MRPL31, IFM1, MRPL24, MRPL23, RPN11, MRPL22, YTA12, MRPL28, HSP78, MRPL27, PHB2, MRPL25, SLS1, RSM25, RSM23, DNM1, RSM22 |
| mitochondrial translation | 4.29E-59 | YML6, MRPL40, MRPS35, MSR1, MRP13, RRF1, MRP51, MRPL36, MRPL37, MRP17, MRPL38, MRPL32, MSK1, MRPL1, MRPL50, MRPL6, MRPL8, MRPL7, MRPS5, MSW1, MRP4, MRPS9, RSM7, MRP7, MRPS8, SLM5, MRPL49, PET123, MSF1, MRP2, MRPS18, RSM18, RSM19, MST1, DIA4, ISM1, MSY1, MRPL20, IMG1, MRPL11, IMG2, MRPL10, MRPL13, MRPL15, MRPL17, MRPL16, MRPL19, NAM9, RSM10, MRF1, PET112, NAM2, MSE1, SWS2, RSM26, MRPS28, MRP20, MRP21, MRPL31, IFM1, MRPL24, MRPL23, MRPL22, MRPL28, MRPL27, MRPL25, SLS1, RSM25, MSD1, RSM23, RSM22 |
| organelle organization | 2.23E-20 | YML6, MRPL40, MRPS35, MDJ1, MRS11, PEX30, MSO1, TOP3, MRP51, MRPL36, MRPL37, MRPL38, MRPL32, HFM1, MGR2, FZO1, SRC1, MRPL50, TOM70, VPS45, MHR1, MSW1, BEM4, MCM22, NUP42, MAD3, PAM17, SLM5, MRPL49, ATP12, PEX27, ATP11, MSF1, MRPS18, MST1, TOM20, LST8, DIA4, MDM34, QRI7, SHE9, MDM35, SRO7, MSE1, SWS2, MRPS28, PNT1, TIM13, CCE1, SAM50, GTS1, MSD1, ZIP1, MSR1, RPO41, MRP13, RRF1, RDI1, PEX4, SAM37, LGE1, PCP1, KAR2, PAA1, MRP17, UGO1, COX18, MGM1, MSK1, MSS2, MRPL1, COX23, MRPL6, ACO1, MRPL8, MRPL7, VAM6, SHY1, MRPS5, HSP60, HIF1, SPO22, MRP4, MRPS9, RSM7, MRP7, MRPS8, APC9, PET123, BCS1, MRP2, SSC1, YME2, RSM18, RSM19, ATG12, MSP1, MIA40, HAT2, AFG3, TPM1, ISM1, MSY1, MRPL20, IMG1, MRPL11, IMG2, MRPL10, MRPL13, MRPL15, MRPL17, PEX17, MRPL16, AFG1, MGE1, MAS1, MRPL19, NAM9, RSM10, MRF1, PEX12, PET112, PEX10, NAM2, ERV1, TCM62, RSM26, MRP20, MRP21, RXT3, TLG2, CBP4, MRPL31, IFM1, MRPL24, MRPL23, RPN11, MRPL22, YTA12, BCK1, MRPL28, MRPL27, HSP78, PHB2, MRPL25, SLS1, RSM25, RFA3, RFA2, DNM1, RSM23, RSM22 |
| cellular component organization | 4.25E-17 | YML6, MRPL40, MRPS35, MDJ1, MRS11, PEX30, MSO1, TOP3, ATG5, MRP51, YTH1, MRPL36, SPO71, MRPL37, MRPL38, ATP23, MRPL32, HFM1, MGR2, FZO1, SRC1, MRPL50, TOM70, VPS45, MHR1, PIM1, MSW1, BEM4, MCM22, NUP42, MAD3, KTR3, PAM17, SLM5, HBS1, MRPL49, ATP12, PEX27, ATP11, MSF1, MRPS18, MST1, TOM20, LST8, DIA4, HRD1, MDM34, FKS3, QRI7, SHE9, SMA1, MDM35, SRO7, MSE1, SWS2, MRPS28, OSH6, PNT1, TIM13, CCE1, SAM50, ADD66, GTS1, MSD1, SPR3, ZIP1, COX11, MSR1, RPO41, NAS6, MRP13, RDI1, RRF1, PEX4, SAM37, LGE1, CUP2, CBT1, KAR2, PCP1, PBA1, PAA1, MRP17, SPS22, GUP1, COX18, UGO1, MGM1, MSK1, DAN2, COX23, MSS2, MRPL1, MRPL6, MCD4, ACO1, MRPL8, VAM6, MRPL7, SHY1, MRPS5, HSP60, HIF1, SPO22, HPF1, MRP4, MRPS9, RSM7, MRP7, MRPS8, APC9, PET123, BCS1, MRP2, SSC1, YME2, RSM18, ATG12, RSM19, MSP1, VPH2, MIA40, HAT2, SPP382, AFG3, TPM1, DSS1, MRPL20, MSY1, ISM1, IMG1, MRPL11, IMG2, MRPL10, MRPL13, MRPL15, DIT1, PEX17, MRPL17, AFG1, MRPL16, MRPL19, MGE1, MAS1, NAM9, MRF1, RSM10, PEX12, PET112, PEX10, NAM2, SCO1, ERV1, CDA1, PPM1, TCM62, CWC23, RSM26, MRP20, MRP21, HSV2, RXT3, TLG2, CBP4, RPB4, |

| | | |
|---|---|---|
| | | MRPL31, IFM1, MRPL24, MRPL23, RPN11, MRPL22, YTA12, BCK1, MRPL28, MRPL27, HSP78, PHB2, MRPL25, SLS1, CYT2, RSM25, RFA3, RFA2, DNM1, RSM23, RSM22 |
| tRNA aminoacylation for mitochondrial protein translation | 3.03E-12 | MSR1, SLM5, MST1, MSF1, MSD1, DIA4, MSW1, MSK1, MSE1, ISM1, MSY1 |
| cellular process | 7.52E-12 | MRPL40, UBP11, MDJ1, SAP4, ECM22, CAK1, UBP16, SLM3, MPD1, TBS1, TRK2, MSS18, EMP47, PEX30, RTA1, MRP51, MTG2, MRPL36, SPO71, MRPL37, AAD15, SPO74, MRPL38, MRPL32, HFM1, OXR1, MGR2, DLD1, SRC1, MRP49, MGR1, MRPL50, TOM70, VPS45, PIM1, MSW1, BEM4, NUP42, SIP2, RRI2, KTR3, MTF2, ARH1, SLM5, HBS1, MRPL49, ATP12, PEX27, ATP11, MSF1, CCC2, PYC1, VBA2, MST1, EUG1, HRD1, ACP1, COY1, SNO3, SNO2, HAP5, MGA2, QRI7, SMA1, SRO7, QRI5, MSE1, SUV3, SGA1, GRX5, UBP5, ARF2, TIM13, CCE1, SAM50, ADD66, ISD11, GTS1, MSD1, COX11, MSR1, HEM2, COX10, NAS6, MLH2, RDI1, SAL1, SAM37, ISA2, HNT3, FRE6, CBT1, CUP2, CIT2, KAR2, HNT1, PBA1, MSC6, PAA1, GUP1, MPH1, MSS51, SPS22, COX18, IAH1, MSS1, DAN2, COX23, MRPL1, MSS2, CDC8, MRPL6, MCD4, RNR3, ACO1, MRPL8, NMA2, VAM6, MRPL7, SHY1, MRPL9, HSP60, SPO22, HPF1, CBS2, NUS1, RSM7, MED8, APC9, PET123, CTM1, BOI1, GYP7, SSC1, HUG1, YME2, DAK2, ATG12, CYM1, MSP1, SPP382, AFG3, BET3, NTA1, BET4, FPR2, MRPL20, MRPL11, MRPL10, MRPL13, MRPL15, PEX17, MRPL17, AFG1, MRPL16, MRPL19, MGE1, MAS1, MRF1, MSS116, PEX12, PET112, PEX10, ERV1, CDA1, TCM62, AAD3, CWC23, PMT3, VTA1, ASK10, NAT2, HSV2, TLG2, EMI2, CBP4, MRPL31, CBP2, IFM1, EMI5, MRPL24, MRPL23, TEX1, MRPL22, BCK1, MRPL28, MRPL27, HSP78, MRPL25, STE3, MHT1, PRE8, AAD4, RFA3, COG1, ABZ1, RFA2, DNM1, ABZ2, YML6, NBP35, MRPS35, DER1, ENB1, VMR1, PET8, QDR1, MRS11, MSO1, TOP3, HXT17, ATG5, CLP1, YTH1, TPT1, HXT13, YUH1, HXT11, HXT12, FZO1, REC104, MHR1, NCE103, XDJ1, MCM22, NFU1, MAD3, PAM17, MCH2, FAD1, PMR1, ADK2, NPL4, FET3, BCH1, MRPS18, MIC17, POL31, FMS1, CUS2, RML2, TOM20, LST8, DIA4, MDM34, SRX1, PYK2, MTQ1, SCS3, CPR6, MSM1, MNS1, BDS1, TAT1, FKS3, SHE9, MDM35, SWS2, MUC1, MRPS28, PET54, OSH6, ARN1, PNT1, CRC1, SCL1, PAD1, SPR3, ZIP1, ALG1, RPO41, MRP13, MNP1, RRF1, PEX4, LGE1, JLP1, RMD9, PCP1, REH1, SOL2, MRP17, SOL1, NTG1, NTG2, UGO1, MGM1, MSK1, PRP46, LHS1, MRPS5, HIF1, IMD2, MAM33, ARL3, MRP4, VID22, COQ3, COQ1, MRPS9, MRP7, MRPS8, APL3, PLM2, BCS1, MRP2, ADD37, RSM18, RSM19, HCS1, VPH2, HUA1, MIA40, ACB1, HAT2, TPM1, STL1, DSS1, MSY1, ISM1, IMG1, PDR8, MTM1, IMG2, LIP2, DIT1, NAM9, PDR1, RSM10, ZWF1, NAM2, MDL2, LPP1, SCO1, RSM28, PPM1, RSM26, MRP20, MRP21, RXT3, RPB4, RPN11, YTA12, PHB2, TDP1, SKM1, SLS1, CYT2, RSM25, ERP3, YET1, BUD5, RSM23, RSM22 |
| protein targeting to mitochondrion | 1.01E-09 | TOM70, MSP1, MIA40, AFG3, HSP60, TOM20, SAM37, MRS11, YTA12, PAM17, HSP78, PCP1, TIM13, MAS1, MGE1, SAM50, SSC1, ERV1 |
| protein localization in mitochondrion | 1.01E-09 | TOM70, MSP1, MIA40, AFG3, HSP60, TOM20, SAM37, MRS11, YTA12, PAM17, HSP78, PCP1, TIM13, MAS1, MGE1, SAM50, SSC1, ERV1 |
| cellular protein metabolic process | 1.26E-09 | YML6, MRPL40, MRPS35, UBP11, MDJ1, ECM22, DER1, CAK1, UBP16, MPD1, ATG5, RTA1, MRP51, MTG2, MRPL36, MRPL37, YUH1, MRPL38, MRPL32, MRP49, MGR1, MRPL50, PIM1, MSW1, XDJ1, NUP42, SIP2, RRI2, KTR3, MTF2, SLM5, HBS1, MRPL49, MSF1, NPL4, FET3, MRPS18, PYC1, MST1, RML2, EUG1, DIA4, HRD1, MTQ1, SCS3, CPR6, MSM1, MNS1, MGA2, MSE1, SWS2, MRPS28, UBP5, SCL1, ADD66, PAD1, MSD1, MSR1, ALG1, MRP13, MNP1, RRF1, PEX4, LGE1, RMD9, PCP1, KAR2, HNT1, MRP17, NTG1, GUP1, UGO1, COX18, MSK1, MRPL1, COX23, MRPL6, MCD4, NMA2, MRPL8, MRPL7, MRPS5, HSP60, MRPL9, HIF1, HPF1, MRP4, VID22, MRPS9, RSM7, NUS1, CBS2, MRP7, MRPS8, APC9, PET123, CTM1, MRP2, SSC1, GYP7, ADD37, RSM18, RSM19, CYM1, ATG12, HUA1, ACB1, HAT2, AFG3, NTA1, FPR2, BET4, DSS1, MRPL20, MSY1, ISM1, IMG1, MRPL11, MTM1, IMG2, LIP2, MRPL10, MRPL13, MRPL15, MRPL17, AFG1, MRPL16, MRPL19, |

| | | |
|---|---|---|
| | | MGE1, MAS1, NAM9, MRF1, RSM10, PET112, PEX10, NAM2, RSM28, PPM1, TCM62, CWC23, RSM26, PMT3, MRP20, MRP21, NAT2, RXT3, CBP4, MRPL31, IFM1, MRPL24, MRPL23, RPN11, MRPL22, YTA12, BCK1, MRPL28, MRPL27, HSP78, PHB2, SKM1, MRPL25, SLS1, CYT2, PRE8, RSM25, RFA3, ABZ1, RFA2, RSM23, RSM22 |
| mitochondrial transport | 2.10E-09 | TOM70, MSP1, MIA40, AFG3, HSP60, TOM20, SAL1, SAM37, MRS11, YTA12, PAM17, HSP78, PCP1, TIM13, MAS1, MGE1, SAM50, MSK1, SSC1, ERV1 |
| protein metabolic process | 4.49E-09 | YML6, MRPL40, NBP35, MRPS35, UBP11, MDJ1, ECM22, DER1, CAK1, UBP16, MPD1, ATG5, RTA1, MRP51, MTG2, MRPL36, MRPL37, YUH1, MRPL38, ATP23, MRPL32, MRP49, MGR1, MRPL50, PIM1, MSW1, XDJ1, NUP42, SIP2, RRI2, KTR3, MTF2, SLM5, HBS1, MRPL49, MSF1, NPL4, FET3, MRPS18, PYC1, MST1, RML2, EUG1, DIA4, HRD1, MTQ1, SCS3, CPR6, MSM1, MNS1, QRI7, MGA2, MSE1, SWS2, MRPS28, UBP5, SCL1, ADD66, PAD1, MSD1, MSR1, ALG1, NAS6, MRP13, MNP1, RRF1, PEX4, ISA2, LGE1, RMD9, PCP1, KAR2, HNT1, MRP17, NTG1, GUP1, UGO1, COX18, MSK1, MRPL1, COX23, MRPL6, MCD4, NMA2, MRPL8, MRPL7, MRPS5, HSP60, MRPL9, HIF1, HPF1, MRP4, VID22, MRPS9, RSM7, NUS1, CBS2, MRP7, MRPS8, APC9, PET123, CTM1, MRP2, SSC1, GYP7, ADD37, RSM18, ATG12, CYM1, RSM19, HUA1, ACB1, HAT2, AFG3, NTA1, BET4, FPR2, DSS1, MRPL20, MSY1, ISM1, IMG1, MRPL11, MTM1, IMG2, LIP2, MRPL10, MRPL13, MRPL15, MRPL17, AFG1, MRPL16, MRPL19, MGE1, MAS1, NAM9, MRF1, RSM10, PET112, PEX10, NAM2, RSM28, PPM1, TCM62, CWC23, RSM26, PMT3, MRP20, MRP21, NAT2, RXT3, CBP4, MRPL31, IFM1, MRPL24, MRPL23, RPN11, MRPL22, YTA12, BCK1, MRPL28, MRPL27, HSP78, PHB2, SKM1, MRPL25, SLS1, CYT2, PRE8, RSM25, RFA3, ABZ1, RFA2, RSM23, RSM22 |
| translation | 9.20E-09 | MRPL40, YML6, MRPS35, ECM22, MTG2, MRP51, MRPL36, MRPL37, MRPL38, MRPL32, MRP49, MRPL50, MSW1, NUP42, MTF2, HBS1, SLM5, MRPL49, MSF1, FET3, MRPS18, MST1, RML2, DIA4, SCS3, MTQ1, MSM1, MGA2, MSE1, SWS2, MRPS28, MSD1, PAD1, MSR1, MRP13, RRF1, MNP1, PEX4, RMD9, HNT1, NTG1, MRP17, UGO1, COX18, MSK1, MRPL1, COX23, MRPL6, MRPL8, MRPL7, MRPL9, MRPS5, HPF1, MRP4, RSM7, CBS2, MRPS9, MRPS8, MRP7, PET123, MRP2, RSM18, RSM19, CYM1, ACB1, ISM1, MSY1, MRPL20, MRPL11, IMG1, MRPL10, IMG2, MTM1, MRPL13, MRPL15, MRPL17, MRPL16, MRPL19, NAM9, RSM10, MRF1, PET112, NAM2, RSM28, RSM26, MRP20, MRP21, CBP4, MRPL31, IFM1, MRPL24, MRPL23, MRPL22, MRPL28, MRPL27, MRPL25, SLS1, RSM25, RSM23, ABZ1, RSM22 |
| intracellular protein transmembrane transport | 1.70E-08 | TOM70, LHS1, MIA40, AFG3, HSP60, TOM20, YTA12, HSP78, PAM17, KAR2, PCP1, SLS1, MGE1, SSC1, ERV1 |
| tRNA aminoacylation | 9.98E-08 | MSR1, MST1, MSW1, DIA4, MSY1, ISM1, MSM1, SLM5, MSF1, PET112, MSD1, NAM2, MSE1, MSK1 |
| amino acid activation | 9.98E-08 | MSR1, MST1, MSW1, DIA4, MSY1, ISM1, MSM1, SLM5, MSF1, PET112, MSD1, NAM2, MSE1, MSK1 |
| tRNA aminoacylation for protein translation | 2.89E-07 | MSR1, MST1, MSW1, DIA4, MSY1, ISM1, MSM1, SLM5, MSF1, MSD1, MSE1, MSK1, NAM2 |
| protein import into mitochondrial intermembrane space | 3.55E-07 | YTA12, PCP1, MIA40, AFG3, HSP60, ERV1 |
| protein import | 6.21E-07 | TOM70, MSP1, MIA40, AFG3, HSP60, TOM20, PEX4, SAM37, MRS11, NUP42, YTA12, PAM17, HSP78, PEX17, PCP1, AFG1, TIM13, MAS1, MGE1, SAM50, PEX12, PEX10, SSC1, ERV1 |
| mitochondrial genome maintenance | 2.21E-06 | MGR2, YME2, MDJ1, RPO41, HSP78, ACO1, MRPL8, MHR1, CCE1, UGO1, QRI7, MGM1 |
| Group I intron splicing | 3.82E-06 | SUV3, PET54, MSS116, NAM2, CBP2, MSS18 |

| | | |
|---|---|---|
| RNA splicing, via transesterification reactions with guanosine as nucleophile | 3.82E-06 | SUV3, PET54, MSS116, NAM2, CBP2, MSS18 |
| mitochondrial membrane organization | 4.68E-06 | MSS2, TOM70, PNT1, TIM13, TOM20, SAM50, MSF1, COX18, SHE9, SAM37, MRS11 |
| aerobic respiration | 1.00E-05 | MRPS35, DLD1, MRPL1, MIC17, COX23, COX11, ACO1, RPO41, MNP1, SHY1, MAM33, DIA4, EMI5, MRPL22, RMD9, CIT2, SLS1, MRF1, BCS1 |
| cellular protein complex assembly | 1.37E-05 | PPM1, COX23, TCM62, NAS6, VPH2, PIM1, SHY1, HSP60, TOM20, CBP4, SAM37, DSS1, CBT1, PEX17, PBA1, ATP12, CYT2, ATP11, ADD66, BCS1, MDM35, SCO1 |
| regulation of mitochondrion organization | 1.89E-05 | HSP78, MHR1, PET54, NTG1, MSS51, PET494 |

**Table S4**. Summary of over-representative biological process in of the significant genes which are positively correlated to *INO2* showing higher than 0.50 in Pearson correlation values.

| Description | p-value | Gene in the test |
|---|---|---|
| lipid biosynthetic process | 4.56E-11 | CPT1, ACC1, INO1, PSD1, INO4, EPT1, CDS1, CKI1, OPI3, FAS1, FAS2, CHO1, HTD2, CHO2, FEN1 |
| phosphatidylcholine biosynthetic process | 2.33E-10 | CPT1, CHO2, PSD1, EPT1, CKI1, OPI3 |
| phosphatidylcholine metabolic process | 1.01E-09 | CPT1, CHO2, PSD1, EPT1, CKI1, OPI3 |
| cellular lipid metabolic process | 2.77E-09 | CPT1, ACC1, INO1, EHT1, PSD1, INO4, EPT1, CDS1, CKI1, OPI3, FAS1, FAS2, CHO1, HTD2, CHO2, FEN1 |
| ethanolamine and derivative metabolic process | 3.22E-09 | CPT1, CHO2, PSD1, EPT1, CKI1, OPI3 |
| phospholipid biosynthetic process | 5.02E-09 | CPT1, CHO1, INO1, CHO2, INO4, PSD1, EPT1, CDS1, CKI1, OPI3 |
| small molecule metabolic process | 8.77E-09 | SPE3, CPT1, INO1, ADH7, EPT1, CKI1, OPI3, SAP30, ARG4, CHO1, GLO2, HTD2, CHO2, URA10, FEN1, FOL1, URA5, ACC1, HIS3, ADO1, SAH1, EHT1, PSD1, SAM2, SET2, FAS1, FAS2, XPT1, NNT1 |
| lipid metabolic process | 2.62E-08 | CPT1, ACC1, INO1, EHT1, PSD1, INO4, EPT1, CDS1, CKI1, OPI3, FAS1, FAS2, CHO1, HTD2, CHO2, FEN1 |
| glycerophospholipid biosynthetic process | 5.50E-08 | CPT1, CHO1, CHO2, PSD1, EPT1, CDS1, CKI1, OPI3 |
| glycerolipid biosynthetic process | 7.48E-08 | CPT1, CHO1, CHO2, PSD1, EPT1, CDS1, CKI1, OPI3 |
| cellular biogenic amine metabolic process | 8.89E-08 | SPE3, CPT1, CHO2, PSD1, EPT1, CKI1, OPI3 |
| small molecule biosynthetic process | 3.81E-07 | SPE3, FOL1, ACC1, URA5, HIS3, INO1, ADO1, EPT1, SAM2, CKI1, FAS1, FAS2, XPT1, ARG4, HTD2, URA10, FEN1 |
| phospholipid metabolic process | 3.92E-07 | CPT1, CHO1, INO1, CHO2, INO4, PSD1, EPT1, CDS1, CKI1, OPI3 |
| organophosphate metabolic process | 5.35E-07 | CPT1, CHO1, INO1, CHO2, INO4, PSD1, EPT1, CDS1, CKI1, OPI3 |
| cellular amino acid derivative metabolic process | 6.63E-07 | SPE3, CPT1, CHO2, PSD1, EPT1, SAM2, CKI1, OPI3 |
| glycerophospholipid metabolic process | 1.68E-06 | CPT1, CHO1, CHO2, PSD1, EPT1, CDS1, CKI1, OPI3 |
| fatty acid biosynthetic process | 2.67E-06 | FAS1, FAS2, ACC1, HTD2, FEN1 |
| glycerolipid metabolic process | 2.92E-06 | CPT1, CHO1, CHO2, PSD1, EPT1, CDS1, CKI1, OPI3 |
| cellular biosynthetic process | 9.56E-06 | SPE3, CPT1, RSF1, RPL22A, DPH5, INO1, INO4, EPT1, CKI1, OPI3, SAP30, ARG4, AGA1, CHO1, KAR4, HTD2, CHO2, NRG2, FEN1, URA10, FOL1, ACC1, URA5, HIS3, ADO1, SAM3, PSD1, CDS1, RPL31A, PRI1, SAM2, SET2, PRM7, FAS1, SSN3, FAS2, XPT1, FIG2, BSC1, TMA20, MOT2 |
| amine metabolic process | 1.36E-05 | SPE3, FOL1, CPT1, HIS3, SAH1, PSD1, EPT1, CKI1, SAM2, OPI3, ARG4, CHO2, UTR2 |
| biosynthetic process | 1.45E-05 | SPE3, CPT1, RSF1, RPL22A, DPH5, INO1, INO4, EPT1, CKI1, OPI3, SAP30, ARG4, AGA1, CHO1, KAR4, HTD2, CHO2, NRG2, FEN1, URA10, FOL1, ACC1, URA5, HIS3, ADO1, SAM3, PSD1, CDS1, RPL31A, PRI1, SAM2, SET2, PRM7, FAS1, SSN3, FAS2, XPT1, FIG2, BSC1, TMA20, MOT2 |
| cellular amine metabolic process | 2.16E-05 | FOL1, SPE3, CPT1, ARG4, HIS3, SAH1, CHO2, PSD1, EPT1, SAM2, CKI1, OPI3 |
| lipid biosynthetic process | 4.56E-11 | CPT1, ACC1, INO1, PSD1, INO4, EPT1, CDS1, CKI1, OPI3, FAS1, FAS2, CHO1, HTD2, CHO2, FEN1 |
| phosphatidylcholine biosynthetic process | 2.33E-10 | CPT1, CHO2, PSD1, EPT1, CKI1, OPI3 |

| | | |
|---|---|---|
| phosphatidylcholine metabolic process | 1.01E-09 | CPT1, CHO2, PSD1, EPT1, CKI1, OPI3 |
| cellular lipid metabolic process | 2.77E-09 | CPT1, ACC1, INO1, EHT1, PSD1, INO4, EPT1, CDS1, CKI1, OPI3, FAS1, FAS2, CHO1, HTD2, CHO2, FEN1 |
| ethanolamine and derivative metabolic process | 3.22E-09 | CPT1, CHO2, PSD1, EPT1, CKI1, OPI3 |
| phospholipid biosynthetic process | 5.02E-09 | CPT1, CHO1, INO1, CHO2, INO4, PSD1, EPT1, CDS1, CKI1, OPI3 |
| small molecule metabolic process | 8.77E-09 | SPE3, CPT1, INO1, ADH7, EPT1, CKI1, OPI3, SAP30, ARG4, CHO1, GLO2, HTD2, CHO2, URA10, FEN1, FOL1, URA5, ACC1, HIS3, ADO1, SAH1, EHT1, PSD1, SAM2, SET2, FAS1, FAS2, XPT1, NNT1 |
| lipid metabolic process | 2.62E-08 | CPT1, ACC1, INO1, EHT1, PSD1, INO4, EPT1, CDS1, CKI1, OPI3, FAS1, FAS2, CHO1, HTD2, CHO2, FEN1 |
| glycerophospholipid biosynthetic process | 5.50E-08 | CPT1, CHO1, CHO2, PSD1, EPT1, CDS1, CKI1, OPI3 |
| glycerolipid biosynthetic process | 7.48E-08 | CPT1, CHO1, CHO2, PSD1, EPT1, CDS1, CKI1, OPI3 |
| cellular biogenic amine metabolic process | 8.89E-08 | SPE3, CPT1, CHO2, PSD1, EPT1, CKI1, OPI3 |
| small molecule biosynthetic process | 3.81E-07 | SPE3, FOL1, ACC1, URA5, HIS3, INO1, ADO1, EPT1, SAM2, CKI1, FAS1, FAS2, XPT1, ARG4, HTD2, URA10, FEN1 |
| phospholipid metabolic process | 3.92E-07 | CPT1, CHO1, INO1, CHO2, INO4, PSD1, EPT1, CDS1, CKI1, OPI3 |
| organophosphate metabolic process | 5.35E-07 | CPT1, CHO1, INO1, CHO2, INO4, PSD1, EPT1, CDS1, CKI1, OPI3 |
| cellular amino acid derivative metabolic process | 6.63E-07 | SPE3, CPT1, CHO2, PSD1, EPT1, SAM2, CKI1, OPI3 |
| glycerophospholipid metabolic process | 1.68E-06 | CPT1, CHO1, CHO2, PSD1, EPT1, CDS1, CKI1, OPI3 |
| fatty acid biosynthetic process | 2.67E-06 | FAS1, FAS2, ACC1, HTD2, FEN1 |
| glycerolipid metabolic process | 2.92E-06 | CPT1, CHO1, CHO2, PSD1, EPT1, CDS1, CKI1, OPI3 |
| cellular biosynthetic process | 9.56E-06 | SPE3, CPT1, RSF1, RPL22A, DPH5, INO1, INO4, EPT1, CKI1, OPI3, SAP30, ARG4, AGA1, CHO1, KAR4, HTD2, CHO2, NRG2, FEN1, URA10, FOL1, ACC1, URA5, HIS3, ADO1, SAM3, PSD1, CDS1, RPL31A, PRI1, SAM2, SET2, PRM7, FAS1, SSN3, FAS2, XPT1, FIG2, BSC1, TMA20, MOT2 |
| amine metabolic process | 1.36E-05 | SPE3, FOL1, CPT1, HIS3, SAH1, PSD1, EPT1, CKI1, SAM2, OPI3, ARG4, CHO2, UTR2 |
| biosynthetic process | 1.45E-05 | SPE3, CPT1, RSF1, RPL22A, DPH5, INO1, INO4, EPT1, CKI1, OPI3, SAP30, ARG4, AGA1, CHO1, KAR4, HTD2, CHO2, NRG2, FEN1, URA10, FOL1, ACC1, URA5, HIS3, ADO1, SAM3, PSD1, CDS1, RPL31A, PRI1, SAM2, SET2, PRM7, FAS1, SSN3, FAS2, XPT1, FIG2, BSC1, TMA20, MOT2 |
| cellular amine metabolic process | 2.16E-05 | FOL1, SPE3, CPT1, ARG4, HIS3, SAH1, CHO2, PSD1, EPT1, SAM2, CKI1, OPI3 |

**Table S5**. Summary of over-representative biological process in of the significant genes which are negatively correlated to *INO2* showing lower than -0.50 in Pearson correlation values.

| Description | p-value | Gene in the test |
|---|---|---|
| intracellular protein transport | 8.36E-10 | MSP1, NSP1, ATG11, PEX6, AFG3, BET4, SEC61, RIC1, RET2, DNF1, ATG5, PCP1, KAR2, AFG1, MAS1, SEC21, VPS35, VPS16, SEC24, PEX10, SEC27, TOM70, VPS45, LHS1, BPH1, HSP60, MON1, SSA2, IMH1, NUP42, APS1, YTA12, HSP78, SAM50, VPS27, SSC1 |
| establishment of protein localization | 2.10E-09 | NSP1, PEX6, OPI1, RET2, ATG5, KAR2, PCP1, VPS16, SRC1, TOM70, VPS45, VAM6, LHS1, HSP60, IMH1, NUP42, SCJ1, ACT1, SEC39, NPL4, SSC1, BCH1, ATG11, MSP1, AFG3, BET4, SEC61, RIC1, CHS5, UBX2, DNF1, AFG1, MAS1, SEC21, VPS35, SEC24, PEX10, SEC27, STV1, BPH1, SSA2, MON1, COG4, APS1, YTA12, ARF2, HSP78, SAM50, VPS27 |
| protein transport | 4.54E-09 | BCH1, NSP1, MSP1, ATG11, PEX6, AFG3, BET4, SEC61, CHS5, RIC1, RET2, UBX2, DNF1, ATG5, PCP1, KAR2, AFG1, SEC21, MAS1, VPS35, VPS16, SEC24, PEX10, SEC27, TOM70, VPS45, LHS1, VAM6, BPH1, HSP60, MON1, SSA2, IMH1, COG4, NUP42, APS1, YTA12, SCJ1, ARF2, HSP78, ACT1, SEC39, SAM50, VPS27, NPL4, SSC1 |
| cellular protein localization | 5.21E-09 | NSP1, MSP1, ATG11, PEX6, AFG3, BET4, SEC61, SPO13, RIC1, RET2, DNF1, ATG5, PCP1, KAR2, AFG1, MAS1, SEC21, VPS35, VPS16, SEC24, PEX10, SEC27, TOM70, VPS45, LHS1, BPH1, HSP60, MON1, SSA2, IMH1, NUP42, APS1, YTA12, YTA7, HSP78, SAM50, VPS27, SSC1 |
| protein localization | 1.34E-08 | NSP1, PEX6, OPI1, RET2, ATG5, KAR2, PCP1, VPS16, SRC1, TOM70, VPS45, VAM6, LHS1, HSP60, IMH1, NUP42, YTA7, SCJ1, ACT1, SEC39, NPL4, SSC1, BCH1, ATG11, MSP1, AFG3, BET4, SEC61, SPO13, RIC1, CHS5, UBX2, DNF1, AFG1, MAS1, SEC21, VPS35, SEC24, PEX10, SEC27, STV1, BPH1, SSA2, MON1, COG4, APS1, YTA12, ARF2, HSP78, SAM50, VPS27 |
| cellular macromolecule localization | 1.36E-08 | NSP1, MSP1, ATG11, PEX6, AFG3, BET4, SEC61, SPO13, RIC1, RET2, DNF1, ATG5, PCP1, KAR2, AFG1, MAS1, SEC21, VPS35, VPS16, SEC24, PEX10, SEC27, TOM70, VPS45, LHS1, BPH1, HSP60, MON1, SSA2, IMH1, NUP42, APS1, YTA12, YTA7, HSP78, SAM50, VPS27, SSC1 |
| macromolecule localization | 2.32E-08 | NSP1, PEX6, OPI1, RET2, PDR11, ATG5, KAR2, PCP1, VPS16, MSK1, SRC1, TOM70, VPS45, VAM6, LHS1, HSP60, IMH1, NUP42, YTA7, SCJ1, ACT1, SEC39, NPL4, SSC1, BCH1, ATG11, MSP1, AFG3, BET4, SEC61, SPO13, RIC1, CHS5, DSS1, UBX2, DNF1, AFG1, MAS1, SEC21, VPS35, SEC24, PEX10, SEC27, STV1, BPH1, MON1, SSA2, SFH5, COG4, APS1, YTA12, ARF2, HSP78, SAM50, VPS27, AUS1 |
| cellular process | 3.40E-08 | APP1, ABC1, MDJ1, SAP4, BNA5, CRH1, NSP1, RET2, TRK2, EMP47, PEX30, RTA1, LCB5, SPO71, AAD15, SPO74, HIR3, MAM1, HFM1, SRC1, DLD1, PAN3, MGR1, TOM70, VPS45, PIM1, UBR2, SLN1, BEM4, FAR11, IMH1, RAD2, NUP42, MPE1, YTA7, ACT1, PCL9, CCC2, PYC1, VBA2, RIC1, RPT6, JEM1, COY1, IZH1, SMA1, SMP3, GDE1, ECM5, SSA2, RPT4, ARF2, UBA1, SAM50, ADD66, MSD1, SPR28, MSR1, HEM2, MLH2, SAL1, SKS1, KAR2, PBA1, FRE2, CUP1-2, CUP1-1, MPH1, GUP1, HST3, RNR3, MCD4, NMA2, VAM6, YRM1, TSA1, HSP60, UFD2, SPO23, RAD50, MSB2, CTM1, BOI1, SSC1, HUG1, YME2, ATG11, MSP1, DAK1, FHL1, APC1, AFG3, SPP382, NTA1, BET3, BET4, SPO13, AFG1, MAS1, THI21, PET112, PEX10, YRF1-5, CWC25, YRF1-6, YRF1-7, AAD3, YRF1-1, PMT3, YRF1-2, YRF1-3, YRF1-4, STV1, SRS2, GPI17, CBP2, COG4, APS1, BCK1, HSP78, AAD4, ABZ1, INO80, VMR1, OPI1, QDR1, YDC1, TOP2, RPN9, ATG5, CLP1, HXT11, HXT12, PDI1, RPN6, FZO1, REC104, XDJ1, MMR1, SEC39, PMR1, NPL4, RAD14, BCH1, |

| | | |
|---|---|---|
| | | SMF1, CHS5, UBX2, SEC21, BDS1, TAT1, SEC24, FKS3, SEC27, MUC1, NIS1, BPH1, DNA2, SCL1, ZIP1, ERO1, ALG1, PEX6, BAP3, SIC1, RSC30, JLP1, PCP1, VPS16, SEY1, RPS23A, UGO1, RPS23B, MGM1, MSK1, RSE1, LHS1, IMD2, DRE2, GPB1, COQ1, SCJ1, HSC82, DSE4, DSE3, SPT6, HAT2, SEC61, KRE5, DSS1, DIT2, DNF1, DIT1, ICT1, PDR1, NAM9, VPS35, ZWF1, NAM2, LPP1, PDR3, SCO1, MDL1, SSM4, MSH5, SSK22, MON1, PHR1, RPN11, YTA12, CRN1, PHB2, TDP1, VPS27 |
| localization | 6.40E-08 | ERO1, NSP1, PEX6, VMR1, BAP3, SAL1, OPI1, QDR1, RET2, TRK2, SKS1, EMP47, PDR11, ATG5, PCP1, KAR2, FRE2, GUP1, HXT11, VPS16, HXT12, UGO1, MSK1, SRC1, TOM70, MCD4, VPS45, LHS1, VAM6, HSP60, YRM1, IMH1, NUP42, MMR1, YTA7, SCJ1, ACT1, SEC39, PMR1, NPL4, CCC2, SSC1, BCH1, MSP1, ATG11, VBA2, AFG3, SMF1, BET3, BET4, SEC61, SPO13, CHS5, RIC1, DSS1, UBX2, DNF1, COY1, AFG1, SEC21, MAS1, VPS35, TAT1, SEC24, PDR5, PEX10, SEC27, SCO1, MDL1, NIS1, STV1, BPH1, MON1, SSA2, SFH5, COG4, APS1, YTA12, CRN1, ARF2, HSP78, PHB2, SAM50, VPS27, AUS1 |
| transport | 1.25E-07 | ERO1, NSP1, PEX6, VMR1, BAP3, SAL1, QDR1, RET2, TRK2, SKS1, EMP47, PDR11, ATG5, PCP1, KAR2, FRE2, GUP1, HXT11, VPS16, HXT12, UGO1, MSK1, TOM70, MCD4, VPS45, LHS1, VAM6, HSP60, YRM1, IMH1, NUP42, SCJ1, ACT1, SEC39, PMR1, NPL4, CCC2, SSC1, BCH1, MSP1, ATG11, VBA2, AFG3, SMF1, BET3, BET4, SEC61, CHS5, RIC1, DSS1, UBX2, DNF1, COY1, AFG1, SEC21, MAS1, VPS35, TAT1, SEC24, PDR5, PEX10, SEC27, SCO1, MDL1, NIS1, STV1, BPH1, MON1, SSA2, SFH5, COG4, APS1, YTA12, ARF2, HSP78, SAM50, VPS27, AUS1 |
| establishment of localization | 1.27E-07 | ERO1, NSP1, PEX6, VMR1, BAP3, SAL1, OPI1, QDR1, RET2, TRK2, SKS1, EMP47, PDR11, ATG5, PCP1, KAR2, FRE2, GUP1, HXT11, VPS16, HXT12, UGO1, MSK1, SRC1, TOM70, MCD4, VPS45, LHS1, VAM6, HSP60, YRM1, IMH1, NUP42, SCJ1, ACT1, SEC39, PMR1, NPL4, CCC2, SSC1, BCH1, MSP1, ATG11, VBA2, AFG3, SMF1, BET3, BET4, SEC61, CHS5, RIC1, DSS1, UBX2, DNF1, COY1, AFG1, SEC21, MAS1, VPS35, TAT1, SEC24, PDR5, PEX10, SEC27, SCO1, MDL1, NIS1, STV1, BPH1, MON1, SSA2, SFH5, COG4, APS1, YTA12, ARF2, HSP78, SAM50, VPS27, AUS1 |
| response to stimulus | 1.50E-07 | MDJ1, MLH2, INO80, OPI1, QDR1, SKS1, RSC30, RTA1, ATG5, KAR2, LCB5, FRE2, CUP1-2, CUP1-1, MPH1, AAD15, SEY1, SLH1, PAU1, DAN4, PAN3, LHS1, TSA1, HSP60, YRM1, SLN1, UFD2, BEM4, FAR11, IMH1, RAD50, GPB1, RAD2, SCJ1, MSB2, HSC82, ACT1, RAD14, SSC1, HUG1, SPT6, DAK1, HAT2, ARR2, ICT1, AFG1, IZH1, PDR1, BDS1, PDR5, ZWF1, PDR3, SCO1, AAD3, MSH5, SSK22, BPH1, SSA2, SRS2, PHR1, DNA2, BCK1, ARF2, HSP78, TDP1, AAD4 |
| intracellular transport | 2.16E-07 | BCH1, NSP1, MSP1, ATG11, PEX6, AFG3, BET3, SAL1, BET4, SEC61, CHS5, RIC1, RET2, DSS1, EMP47, DNF1, ATG5, COY1, PCP1, KAR2, AFG1, SEC21, MAS1, VPS35, VPS16, SEC24, PEX10, MSK1, SEC27, TOM70, VPS45, LHS1, BPH1, HSP60, MON1, SSA2, IMH1, COG4, APS1, NUP42, YTA12, HSP78, ARF2, ACT1, SEC39, SAM50, VPS27, SSC1 |
| telomere maintenance via recombination | 2.61E-07 | YRF1-5, YRF1-6, YRF1-7, YRF1-1, YRF1-2, YRF1-3, YRF1-4, RAD50 |
| intracellular protein transmembrane transport | 2.75E-07 | YTA12, TOM70, HSP78, PCP1, KAR2, LHS1, AFG3, HSP60, SEC61, SSA2, SSC1 |
| cellular localization | 3.33E-07 | NSP1, PEX6, SAL1, RET2, EMP47, ATG5, KAR2, PCP1, VPS16, MSK1, TOM70, VPS45, LHS1, HSP60, IMH1, NUP42, YTA7, MMR1, ACT1, SEC39, PMR1, SSC1, BCH1, ATG11, MSP1, AFG3, BET3, BET4, SEC61, SPO13, RIC1, CHS5, DSS1, UBX2, DNF1, COY1, AFG1, MAS1, SEC21, VPS35, SEC24, PEX10, SEC27, BPH1, MON1, SSA2, COG4, APS1, YTA12, CRN1, ARF2, HSP78, PHB2, SAM50, VPS27 |
| establishment of localization in cell | 3.55E-07 | NSP1, PEX6, SAL1, RET2, EMP47, ATG5, KAR2, PCP1, VPS16, MSK1, TOM70, VPS45, LHS1, HSP60, IMH1, NUP42, ACT1, SEC39, PMR1, SSC1, BCH1, ATG11, MSP1, AFG3, BET3, BET4, SEC61, RIC1, CHS5, DSS1, UBX2, DNF1, COY1, AFG1, MAS1, SEC21, VPS35, SEC24, PEX10, SEC27, BPH1, SSA2, MON1, COG4, APS1, YTA12, ARF2, HSP78, SAM50, VPS27 |
| protein targeting | 8.88E-07 | ATG11, MSP1, NSP1, AFG3, PEX6, BET4, SEC61, ATG5, KAR2, PCP1, AFG1, MAS1, VPS16, PEX10, TOM70, VPS45, LHS1, HSP60, |

| | | |
|---|---|---|
| | | SSA2, MON1, NUP42, YTA12, HSP78, SAM50, VPS27, SSC1 |
| proteasome assembly | 2.67E-06 | RPN9, RPT4, HSC82, PBA1, ADD66, DSS1, RPN6, RPT6 |
| cellular component organization | 3.55E-06 | APP1, MDJ1, CRH1, NSP1, INO80, RET2, TOP2, RPN9, PEX30, ATG5, SPO71, HIR3, MAM1, HFM1, RPN6, SRC1, FZO1, TOM70, VPS45, PIM1, BEM4, NUP42, MPE1, MMR1, ACT1, SEC39, RAD14, CHS5, RPT6, JEM1, SEC24, FKS3, SMA1, ECM5, BPH1, DNA2, RPT4, SAM50, ADD66, MSD1, SPR28, ZIP1, MSR1, PEX6, RSC30, KAR2, PCP1, PBA1, GUP1, VPS16, SEY1, UGO1, MGM1, MSK1, RSE1, HST3, MCD4, VAM6, HSP60, DRE2, RAD50, MSB2, HSC82, SSC1, YME2, ATG11, MSP1, SPT6, APC1, AFG3, SPP382, HAT2, KRE5, SPO13, DSS1, DIT2, DNF1, DIT1, AFG1, MAS1, NAM9, PET112, PEX10, NAM2, SCO1, YRF1-5, YRF1-6, YRF1-7, YRF1-1, YRF1-2, YRF1-3, YRF1-4, RPN11, YTA12, BCK1, HSP78, CRN1, PHB2 |
| mitochondrial transport | 5.57E-06 | YTA12, TOM70, HSP78, MSP1, PCP1, MAS1, AFG3, HSP60, SAM50, SAL1, MSK1, SSC1 |
| proteolysis | 8.21E-06 | MDJ1, YPS5, APC1, AFG3, RPT6, DSS1, RPN9, JEM1, UBX2, KAR2, AFG1, MAS1, ECM29, RPN6, SSM4, MGR1, PIM1, UFD2, GPB1, RPN11, RPT4, YTA12, UBA1, SCJ1, PRD1, SCL1, ADD66, NPL4 |
| proteolysis involved in cellular protein catabolic process | 8.47E-06 | SSM4, MDJ1, MGR1, APC1, PIM1, UFD2, DSS1, RPT6, GPB1, RPN9, JEM1, RPN11, UBX2, RPT4, SCJ1, KAR2, AFG1, SCL1, ADD66, NPL4, RPN6 |
| protein catabolic process | 1.05E-05 | MDJ1, PYC1, APC1, AFG3, NTA1, RPT6, DSS1, YDC1, RPN9, JEM1, UBX2, KAR2, AFG1, ECM29, RPN6, SSM4, MGR1, NMA2, PIM1, UFD2, GPB1, RPN11, RPT4, YTA12, SCJ1, SCL1, ADD66, NPL4 |
| response to stress | 1.35E-05 | MDJ1, SPT6, DAK1, HAT2, MLH2, INO80, OPI1, RSC30, ATG5, RTA1, ICT1, KAR2, LCB5, CUP1-2, AFG1, CUP1-1, MPH1, SEY1, ZWF1, PAU1, PAN3, DAN4, MSH5, LHS1, SSK22, HSP60, TSA1, SLN1, UFD2, SRS2, SSA2, PHR1, IMH1, RAD50, DNA2, RAD2, BCK1, SCJ1, HSP78, ARF2, MSB2, TDP1, HSC82, ACT1, AAD4, RAD14, SSC1, HUG1 |
| protein localization in organelle | 1.63E-05 | TOM70, NSP1, MSP1, LHS1, AFG3, HSP60, SEC61, SSA2, NUP42, YTA12, HSP78, KAR2, PCP1, MAS1, VPS35, SAM50, VPS27, SSC1 |
| protein targeting to mitochondrion | 1.86E-05 | YTA12, TOM70, HSP78, MSP1, PCP1, MAS1, AFG3, HSP60, SAM50, SSC1 |
| protein localization in mitochondrion | 1.86E-05 | YTA12, TOM70, HSP78, MSP1, PCP1, MAS1, AFG3, HSP60, SAM50, SSC1 |
| homeostatic process | 2.34E-05 | YRF1-5, YRF1-6, YRF1-7, YRF1-1, YRF1-2, YRF1-3, VPS45, STV1, YRF1-4, TSA1, SMF1, TRK2, RAD50, DNA2, EMP47, HSC82, IZH1, VPS35, VPS16, PMR1, PDI1, CCC2, SSC1, SCO1 |
